# Impact of Combination Rules, Level of Theory, and Potential Function on the Modeling of Gas- and Condensed-Phase Properties of Noble Gases

**DOI:** 10.1021/acs.jctc.3c01257

**Published:** 2024-03-13

**Authors:** Kristian Kříž, Paul J. van Maaren, David van der Spoel

**Affiliations:** Department of Cell and Molecular Biology, Uppsala University, Box 596, Uppsala SE-75124, Sweden

## Abstract

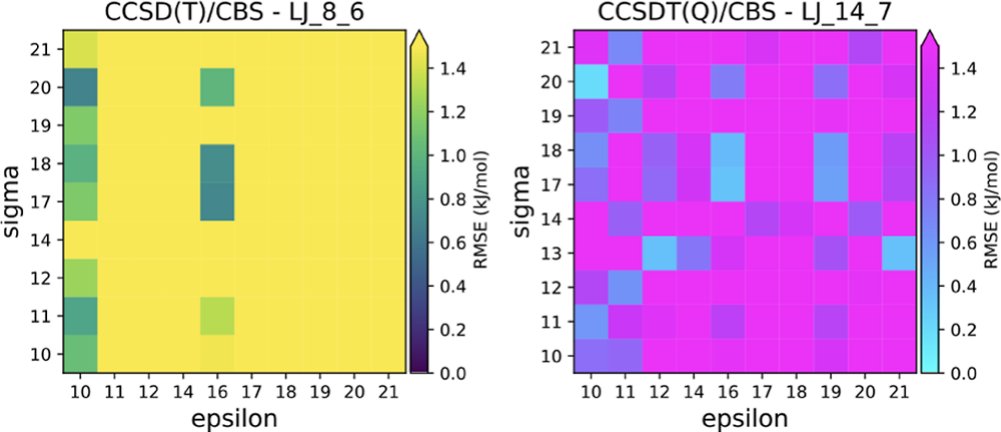

The systems of noble gases are particularly instructive for molecular modeling due to the elemental nature of their interactions. They do not normally form bonds nor possess a (permanent) dipole moment, and the only forces determining their bonding/clustering stems from van der Waals forces—dispersion and Pauli repulsion, which can be modeled by empirical potential functions. Combination rules, that is, formulas to derive parameters for pair potentials of heterodimers from parameters of corresponding homodimers, have been studied at length for the Lennard-Jones 12-6 potentials but not in great detail for other, more accurate, potentials. In this work, we examine the usefulness of nine empirical potentials in their ability to reproduce quantum mechanical (QM) benchmark dissociation curves of noble gas dimers (He, Ne, Ar, Kr, and Xe homo- and heterodimers), and we systematically study the efficacy of different permutations of combination relations for each parameter of the potentials. Our QM benchmark comprises dissociation curves computed by several different coupled cluster implementations as well as symmetry-adapted perturbation theory. The two-parameter Lennard-Jones potentials were decisively outperformed by more elaborate potentials that sport a 25–30 times lower root-mean-square error (RMSE) when fitted to QM dissociation curves. Very good fits to the QM dissociation curves can be achieved with relatively inexpensive four- or even three-parameter potentials, for instance, the damped 14-7 potential (Halgren, *J. Am. Chem. Soc.***1992,***114,* 7827–7843), a four-parameter Buckingham potential (Werhahn et al., *Chem. Phys. Lett.***2015,***619,* 133–138), or the three-parameter Morse potential (Morse, *Phys. Rev.***1929,***34,* 57–64). Potentials for heterodimers that are generated from combination rules have an RMSE that is up to 20 times higher than potentials that are directly fitted to the QM dissociation curves. This means that the RMSE, in particular, for light atoms, is comparable in magnitude to the well-depth of the potential. Based on a systematic permutation of combination rules, we present one or more combination rules for each potential tested that yield a relatively low RMSE. Two new combination rules are introduced that perform well, one for the van der Waals radius σ_*ij*_ as  and one for the well-depth ϵ_*ij*_ as . The QM data and the fitted potentials were evaluated in the gas phase against experimental second virial coefficients for homo- and heterodimers, the latter of which allowed evaluation of the combination rules. The fitted models were used to perform condensed phase molecular dynamics simulations to verify the melting points, liquid densities at the melting point, and the enthalpies of vaporization produced by the models for pure substances. Subtle differences in the benchmark potentials, in particular, the well-depth, due to the level of theory used were found here to have a profound effect on the macroscopic properties of noble gases: second virial coefficients or the bulk properties in simulations. By explicitly including three-body dispersion in molecular simulations employing the best pair potential, we were able to obtain accurate melting points as well as satisfactory densities and enthalpies of vaporization.

## 1 Introduction

Systematic design of empirical potential functions for molecular simulations is a long-standing goal in physical chemistry. What mathematical expression best reproduces relevant experimental properties has been an open question for 50 years.^1−3^ Today, most classical, nonpolarizable, force fields still employ a combination of the Coulomb and Lennard-Jones potentials^4^ in their Hamiltonian. Already in the 1960s, researchers worked on alternative potentials, however. For instance, Konowalow and colleagues used a Morse potential^5^ as well as a modified Buckingham potential.^6^ Munn compared energy functions with the purpose of reproducing the argon second virial coefficient and concluded that accurate potentials differed substantially from the Lennard-Jones potential.^7^ More recently, a number of papers have pointed out issues with the Lennard-Jones potential or shown that other functional forms yield more accurate predictions for other compounds as well.^8−12^

The Lennard-Jones (12-6) potential^4^ involves two parameters, the van der Waals radius σ and the well-depth ϵ (eq 1). Typically, the parameters are taken to be properties of the atom while interactions between different atoms are modeled by combining atomic parameters into atom-pair parameters, i.e. σ_*ij*_ = *f*(σ_*i*_, σ_*j*_) and ϵ_*ij*_ = *g*(ϵ_*i*_, ϵ_*j*_). The use of these so-called combination rules reduces the amount of parameters for N atom types to a linear  rather than quadratic  problem, which is crucial to make parameter search tractable and to prevent overfitting.^3,13^ What functions *f* and *g* give best result for systems combining multiple different atom types has been studied in depth for the Lennard-Jones potential.^14−18^ For other van der Waals potentials, the number of studies is more limited.^19−21^ Since there is ample evidence that the Lennard-Jones is unable to accommodate the repulsive part of the potential faithfully,^8−12,22,23^ it is important to establish (a) what functional forms are better suited to model both Pauli repulsion and dispersion interactions and (b) how atomic parameters can be converted into atom-pair parameters for those functional forms.

It is well established that the accuracy that can be obtained with two body potentials is limited. There are quite a few studies applying three-body terms, ab initio MD, and in some cases even quantum particles and such models reproduce experimental data more accurately than two-body potentials.^24−34^ It is not the intention of this work, however, to propose models that are better than what the state of the art is but rather to derive principles and protocols that can be generalized to molecular compounds. Therefore, we evaluate 9 different pair potentials with 2–5 parameters by fitting the parameters to the potential computed using quantum chemistry for homodimers of noble gases (He–Xe). Then, we evaluate which combination rules best reproduce the quantum chemistry potential for heterodimers. The main focus of this work is to analyze what potentials and combination rules can be applied to macroscopic systems and evaluate the models. We therefore compute both gas- and condensed-phase properties. The former is done by the calculation of second virial coefficients and comparison with experimental data. For the investigation of the latter, we perform a series of MD simulations of monoatomic systems and evaluate melting points, liquid densities, and the enthalpies of vaporization at the melting point. Finally, we add the Axilrod–Teller three-body dispersion^35^ term to one of the best pair potentials and re-evaluate condensed phase properties.

## 2 Theory

Below, we first introduce the van der Waals potentials used and then the combination rules tested.

### 2.1 van der Waals Potential Functions

There are numerous expressions for energy functions governing dispersion and repulsion. In this work, we consider three forms of Lennard-Jones potential—power functions, a Morse potential and four variants of Buckingham functions, featuring an exponential part that is physically more justified for the repulsive component of the potential.^36^ The following equations describe the pair interaction between the respective elements, with the *r* being their separation in space, and the parameters are pair-characteristic ones (discussion on combination rules, see in further sections).

The Lennard-Jones 12-6 potential^4^ is given by
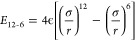
1with σ related to the van der Waals radius of the atom and ϵ the well-depth. The 8-6 potential^37^ is given by
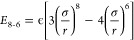
2with σ corresponding to the position of the energy minimum and ϵ the well-depth. The buffered 14-7 potential^38,39^
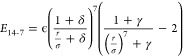
3where γ and δ are dimensionless numbers that were originally shared for all elements. However, in this work, we treat γ and δ as free-atom-specific parameters subject to optimization and combination rules.

For the Morse potential,^21,40^ we use
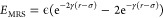
4

The original Buckingham potential,^36^ given by
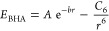
5has three parameters. *A* has the dimension of energy and *b* is a reciprocal length, while *C*_6_ represents the force constant for the attractive dispersion energy.

An alternative way of formulating the Buckingham potential^41^ is
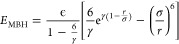
6where ϵ is the well-depth σ reflects the position of the minimum and γ is again a dimensionless constant. Although mathematically identical to eq 5, we will show below that the potentials behave differently when combination rules are applied. The form of this potential was altered by Wang and co-workers^9^ to remove the unphysical behavior at *r* = 0. We have used this form in a transferable model for alkali halides^10^ and applied it to study properties of these materials in all phases.^42−44^ The functional form is
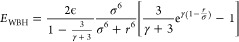
7

Furthermore, we consider a generalized 4-parameter Buckingham potential with an adjustable long-range attraction^45^
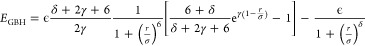
8where γ and δ again are dimensionless constants.

Finally, we use the Tang–Toennies potential^46^ containing five parameters in the dimensionless notation introduced by Wei et al.^20^
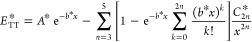
9where *x* = *r*/*R*_e_ and *R*_e_ is the equilibrium distance, A* = *A*/*D*_e_ and *D*_e_ the dissociation energy, and *C*_2n_^*^ = *C*_2*n*_/(*D*_e_*R*_e_^2*n*^). The final energy *E*_TT_ is obtained after multiplying eq 9 by *D*_e_. Wei and co-workers argued that by making the parameters dimensionless, the potential parameters become very similar and therefore it becomes easier to apply combination rules,^20^ and we follow this approach for the TT potential in this work.

### 2.2 Combination Rules for van der Waals Potentials

Combination rules reduce the number of parameters required for the pairwise potentials introduced above because the parameters describing the interaction between dissimilar *X*–*Y* atoms are reconstructed from parameters of homodimers *X*–*X* and *Y*–*Y*. In this way, it is necessary only to fit atomic parameters on data for homodimers. Different sets of mathematical expressions were considered, as detailed below.

The two simplest expressions that have historically been used as combination rules are the geometric^47^ and arithmetic^48^ averages, respectively
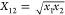
10
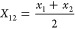
11where *x*_1_ and *x*_2_ are the atomic parameters. Both of these rules can in principle be applied to all parameters in the van der Waals potentials described above, and the rules are well-behaved mathematically.

Hogervorst introduced a set of combination rules for 12-6 Lennard-Jones and exp-6, modified Buckingham, potential (eq 6).^14^ He proposed using eq 12 for ϵ for both potential forms. For the σ of 12-6 potential (eq 1), the arithmetic mean (eq 11) was used. In the case of the modified Buckingham potential (eq 6), expression 13, which depends on the combined parameters ϵ_1,2_ and γ_1,2_, was advocated for the σ, along with arithmetic mean for γ.
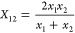
12
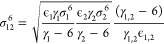
13where γ_1,2_ is used eq 11 and ϵ_1,2_eq 12. It should be noted that eq 12 is ill-behaved if both *x*_1_ and *x*_2_ are zero, while eq 13 is ill-behaved if either γ_1,2_ or ϵ_1,2_ is zero. Yang et al.^21^ introduced an expression for the Morse potential (eq 4), using eq 12 for ϵ, while eq 14 is used for σ and γ.
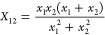
14

The expression for γ proposed by Mason^49^ for the exp-6 potential has the form
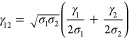
15

Waldman and Hagler^16^ introduced expressions 16 for ϵ and 17 for σ to reproduce experimental well-depths and interaction distances.
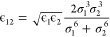
16
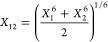
17

Although eq 17 was devised for σ, we have evaluated it for other parameters here as well, hence the notation with *X*. Qi and co-workers advocated the use of buffered 14-7 Lennard-Jones potential (eq 3) due to Halgren,^39^ alongside combination expressions 16 for ϵ and 18 for σ^38^
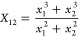
18

A further relation, the harmonic mean rule, was proposed by Halgren^39^
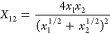
19

Finally, we introduce two new combination rules that we have not seen published previously. Since the σ in most potentials can be interpreted as a van der Waals radius, we include a relation averaging third powers, corresponding to an atomic volume
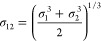
20

In addition, we applied the following rule for, in particular, ϵ since it yields an *X*_12_ that is smaller than the geometric one (eq 10)
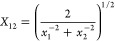
21

The combination relations described above were permuted with each other into new combination rules. In this way, relations that depend on only one parameter type were used for any parameter. Relations depending on multiple parameters were used only for the specific parameter type combination they depend on (e.g., eq 16 was only used for ϵ, using homodimer ϵ and σ). In our previous work on alkali halides,^10^ we used combination rules according to eq 13 for σ, eq 12 for ϵ, and eq 11 for γ with the Wang–Buckingham potential (eq 7).

## 3 Methods

### 3.1 Dimer Dissociation Energy Curves

High-level quantum mechanical (QM) data were used for the construction of dissociation energy curves of noble gas dimers, which were used for fitting of potentials with subsequent combination rules evaluation and force field training as well as for the second virial coefficient calculations.

For all 15 dimers of noble gas atoms from helium to xenon, the dissociation curve was scanned by steps of 0.1 up to 30 Å (QM computations that failed at very short separations were discarded). The interaction energies for dimers at the points of the dissociation curve were computed at the coupled cluster CCSD(T) level with triple and quadruple ζ, from which an extrapolation to the complete basis set (CBS) using the Helgaker scheme^50,51^
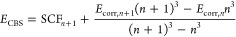
22was performed for the attainment of the “gold standard” level of computational chemistry.^52^*n* denotes the basis set size, using augmented correlation consistent triple to quadruple ζ (aug-cc-pVnZ, n = T, Q) basis sets^53^ for the extrapolation. *E*_corr_ is the difference between the SCF and the CCSD(T) at their respective basis. The frozen-core treatment was omitted (except for pseudopotential use) to allow for the correlating of subvalence orbitals and to facilitate the better, core–valence-weighted^54^ version of the basis set being used for Kr and Xe. Pseudopotentials for the latter elements were used to account for relativistic effects^55^ (aug-cc-pwCVnZ-PP). A counterpoise correction was applied for the basis set superposition error treatment.^56^ The quantum chemistry calculations were performed using the Psi4 suite.^57^

Previously published pair potentials at even higher levels of theory were obtained for helium^58^ neon,^28^ argon,^59^ krypton,^60^ and xenon.^61^ Potentials for heterodimers have been published as well for He–Ne, He–Ar and Ne–Ar,^62^ for He–Kr,^60^ for interaction of Xe with He, Ne, Ar,^63^ as well for interactions of Kr with Ne, Ar, and Xe.^64^ Those are referred to as CCSDT(Q)/CBS in what follows, but for precise details of these calculations, we refer the reader to the original papers.

To examine the usefulness of less-demanding computational approaches, we also performed dimer energy calculations using the SAPT2 + (CCD)δMP2 method.^65^ The symmetry adapted perturbation theory (SAPT) energy determines the interaction energy between two monomers perturbatively and is free of basis set superposition error.^66^ This SAPT variety, combined with the aug-cc-pVTZ basis set, was found to be the most accurate among SAPT methods when tested on “gold standard” benchmark databases of interaction energies.^65^ For a review of using SAPT calculations for force field development, see McDaniel and Schmidt.^67^ However, unlike these authors,^65^ we have additionally employed a larger basis set, quadruple ζ, since it was computationally tractable for our small model systems. The same setup regarding basis set and (not) freezing orbitals as that for CCSD(T) calculations was used for SAPT as well.

### 3.2 Derivation of Empirical Potentials

On the benchmark data of noble gas dimers, a fit of different potential energy functions was done using the Scientific Python module as detailed in the Supporting Information. To perform the fits, energy cutoff were applied on both the repulsive part of the potential and the long tail, limiting the data to an area around the equilibrium but, as is described below, the whole curve was used for determination of second virial coefficients. The reference area of the dissociation curve that was used for parameter fitting had an upper cutoff of 20 kJ/mol and lower cutoff of 10% of the respective energy minimum. Although it is possible to fit and derive more complicated analytical potentials in the limit of the distance going to zero,^68−70^ the purpose of this work is rather to derive combination rules for more affordable potentials. Once the optimal parameters for homodimers were determined by the fits, heterodimer parameters can be generated using combination rules. The energy curves based on combination rules can then be compared to the heterodimer energy from QM. This allowed an exhaustive search for the optimal combining rule (permutation of combination equations for each parameter) for each of the respective van der Waals potential functions. For the case of the Tang–Toennies potential (eq 9), we used the position and depth of the potential well as presented by Wei et al.^20^ as constants in the fitting and only adjusted the five dimensionless quantities. This implies that this potential is applicable only to mixtures of substances where details of the mixed interaction are known beforehand.

The treatment of many-body effects cannot be neglected for noble gases.^71^ For the molecular simulations (see section below), we include the leading term of a three-body potential, the triple dipole potential, as introduced by Axilrod and Teller^35^
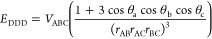
23where A, B and C indicate different atoms—in our case the same atom types, *r*, are corresponding distances between atom pairs and θ are angles between the three atoms. The coefficients *V*_ABC_ were taken from ref (72). This three body potential has been shown to be an adequate measure to account for the three-body nonadditive effects in noble gases.^71^ The potential has been included only to the force field trained at the highest level of theory, with the LJ14-7 potential (eq 3), which is among the ones that faithfully reproduce the dissociation curves.

### 3.3 Second Virial Coefficients

Second virial coefficients *B*_2_(*T*) represent the pairwise potential part of the deviation from the ideal gas law.^73^ These were computed, including quantum corrections to the third order, as described in detail by Bich and co-workers.^74^
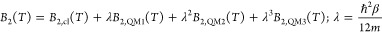
24
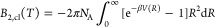
25
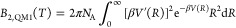
26
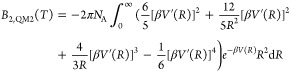
27
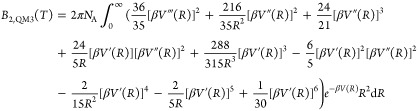
28where *V* is the pair potential, *R* is the interatomic separation, and *m* is the atom mass. This “semiclassical” approach that was used to compute second virial coefficients is suitable for all noble-gas pairs except He–He.^75,76^ For the He dimer, a still more advanced treatment is needed.^77^ Therefore, we will not consider the second virial coefficients of the He dimer in this work. *B*_2_(*T*) for both the QM derived dissociation curves and the analytical potentials were calculated by direct numerical integration where the first, second, and third derivatives of the energy functions needed to compute the quantum corrections were determined from a cubic-spline interpolation. Integration was done to the extent of the QM data set (30 Å for QM results presented here). Since the second virial coefficient is sensitive to the spacing of data points, in particular around the minimum and in particular the quantum corrections, the QM data were interpolated using a cubic spline, corresponding to an effective spacing of 0.025 Å between points. For the analytical potentials, no interpolation was needed, but derivatives were computed using cubic splines. Experimental data for second virial coefficients were taken from Landolt-Börnstein,^78^ with a correction for an error in the Ar–Xe coefficient from the original data.^79−81^

### 3.4 Molecular Simulations

Bulk simulation boxes were constructed by generating a face-centered cubic lattice with lattice parameters corresponding to each respective noble gas atom, extending 6 layers in each axis, accounting for 864 atoms. The cubic boxes were melted using a high-temperature simulation and grafted onto a corresponding not-melted box, creating a rectangular biphasic crystal–liquid system of 1728 atoms with an interface in the middle, corresponding to 12 times the lattice parameter in the longer dimension. Melting points were determined from a temperature scan in steps of 1 K around the experimental melting point of these solid–liquid boxes.^43,82−85^ The density of the liquid in the last 20% of the simulation was plotted against the temperature. A discontinuity in the density was taken as an indication of the melting point, which was then confirmed by a visual inspection. The first temperature at which the whole simulated box (rectangle) visibly melted within the simulation duration was considered to be the melting point. This definition provides a clear measure but sets the melting point to the upper-bond limit. Provided enough simulation time, a box could have attained the single-phase equilibrium (melted) at lower temperature, introducing a small uncertainty, which could have accounted for lowering of melting points by 0–2 K. The corresponding average potential energy of pure liquid boxes was used for the calculation of Δ_vap_*H*. The parameters for force fields were trained on the corresponding benchmark dissociation curves (20 kJ/mol) and the absolute values of 10% of the energy minimum as the upper and lower thresholds, respectively (see Methods). Simulations were performed using the OpenMM^86^ software. They encompassed 250,000 steps of 4 fs—totaling 1 ns, after the minimization and equilibration steps, using the particle mesh Ewald method^87^ for long-range van der Waals interactions. A cutoff of 1 nm was used for the van der Waals functions (including the three body dispersion). Temperature was controlled using the Nosé–Hoover thermostat,^88,89^ and pressure was controlled using a Monte Carlo barostat^90^ in the direction perpendicular to the initial crystal–liquid interface (however, an isotropic barostat was applied for systems used for solid density estimates). For the simulations with and without Teller–Axilrod potential with LJ14-7 potential presented in Table 5, the temperature scan was done in steps of 0.5 K, with a simulation length of 2 ns or more, until only a single phase prevailed in the box. This reduces the previously given approximate error in the melting points estimate to 0.5 K. The simulation box was relaxed in the two dimensions not subject to the anisotropic barostat at the melting temperature prior to the production simulation. The OpenMM interface was used with a custom script implementing alternative van der Waals potentials in direct space, while for the reciprocal space part, a *r*^–6^ dispersion term was used with the force constant matching that of the alternative potentials used. This script is available from github,^91^ see next section.

The average potential energy per atom *E*_pot_(*l*) of the last 20% of simulations at the respective melting points was used for the calculation of heat of vaporization using
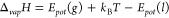
29where *k*_B_ is Boltzmann’s constant. In our models, the potential energy in the gas phase *E*_pot_(*g*) is 0 kJ/mol.

### 3.5 Reproducibility

To allow others to reproduce the work presented here or to extend it, we share the code and inputs on github.^91^ This includes experimental data for the second virial coefficients (see above) and scripts to reproduce previous papers by Hellmann et al.,^58^ Wei et al.,^20^ Jäger and Bich,^92^ as well as Sheng et al.^68^ Force field files for OpenMM including three-body dispersion are provided, as well.

## 4 Results and Discussion

### 4.1 Dissociation Curves

Dissociation curves of noble gas dimers were computed at five different levels of theory, and they are compared to high-quality data—CCSDT(Q), published elsewhere^28,58−64^ (Figure S1). Understandably, SAPT and CCSD(T) computed using the augmented triple ζ (TZ) basis set produced the most shallow dissociation curves. The difference in well-depth between CCSD(T) in TZ and the highest level of theory considered here, CCSDT(Q) extrapolated to the CBS limit, ranged from 0.021 kJ/mol [about 23% of CCSDT(Q)] for the helium dimer to 0.61 kJ/mol (about 26%). The use of quadruple ζ already yields a significant improvement (increase) in well-depth—approximately halfway to the extrapolated CBS methods. The post-CCSD(T)/CBS treatment including relativistic corrections [CCSDT(Q)/CBS] deepened the potentials with respect to CCSD(T)/CBS slightly for the helium dimer, but for the other noble gas dimers, the CCSDT(Q)/CBS potential was a bit less attractive than CCSD(T)/CBS (compare Tables S45 to S37).

The dissociation curves were used for deriving empirical potential functions as well as for an analysis of combination rules that were validated by comparing computed to experimental second virial coefficients. Finally, we report macroscopic observables from bulk simulations of monatomic systems for the force field trained on the dissociation curves of homodimers.

### 4.2 Analytical Fits

Table 1 shows the average root-mean-square error (RMSE) of different potentials fitted to QM data at different levels of theory. The general picture that emerges shows that neither the Lennard-Jones 12-6 nor the 8-6 potential represent the dissociation curves well. More complicated potentials, with more free parameters, get much closer to the reference data. In particular, the generalized Buckingham (eq 8), the buffered 14-7 (eq 3), and the Tang–Toennies (eq 9) potentials yielded very low RMSE. The differences between levels of theory affect the quality of the fit only slightly but not systematically. An analysis of the effect of different energy thresholds and QM methods is given below.

**Table 1:** RMSE (kJ/mol) from Direct Analytical Fit of Potentials to Quantum Chemical Data for Different Levels of Theory^a^

level of theory	LJ12-6	LJ8-6	WBH	MBH	MRS	BHA	GBH	LJ14-7	TT
# params	2	2	3	3	3	3	4	4	5
SAPT/TZ	0.259	0.235	0.065	0.023	0.036	0.023	0.009	0.009	0.011
SAPT/QZ	0.264	0.254	0.075	0.028	0.036	0.028	0.010	0.011	0.008
CCSD(T)/TZ	0.292	0.241	0.070	0.028	0.027	0.028	0.010	0.008	0.006
CCSD(T)/QZ	0.285	0.258	0.079	0.033	0.028	0.033	0.012	0.009	0.007
CCSD(T)/CBS	0.293	0.277	0.090	0.040	0.031	0.040	0.014	0.015	0.011
CCSDT(Q)/CBS	0.238	0.255	0.078	0.033	0.025	0.033	0.012	0.007	0.005

a Repulsive energy cutoff 20 kJ/mol, long range cut at 10% of the well-depth.

Tables S2–S7 list the RMSE from fitting the nine different analytical potentials to all levels of theory applied here and specific for each of the atom pairs studied. Table S6 shows the results for the “gold-standard” level of theory, CCSD(T)/CBS. Aside from Lennard-Jones 12-6 and 8-6, the potentials were able to reproduce the dissociation curves well, within 0.1 kJ/mol of RMSE. The Tang–Toennies 5-parameter potential delivered the best fit of 0.01 kJ/mol, followed closely by the buffered Lennard-Jones 14-7 and generalized Buckingham potentials. Note that the results for the Buckingham and the Modified Buckingham potentials are the same since these potentials are mathematically identical, given the proper parameter conversion. In contrast to what is expected based on theory, the C8 term in the TT potential (eq 9) converges to zero except for dimers including Neon (e.g., Tables S46 and S36). It is well-known that very high levels of theory are needed to accurately predict dispersion coefficients^93^ or indeed to even get the position of the energy minimum of the neon dimer correct.^94^ This is why other studies^20,68^ rely on dispersion coefficients from time-dependent many-body perturbation theory methods.^95^

#### 4.2.1 Energy Cutoffs for Fitting

Increasing the upper-energy threshold from 20 kJ/mol—including more high energy points—deteriorated the fit slightly (Table S1), but it was still very good for most potentials. The Lennard-Jones two parameter potentials both have difficulties accommodating the repulsive part of the dissociation curve. In order to lower the contribution to the RMSE from high-energy points, the fitted potentials often underestimated the well-depth. The 8-6 potential additionally overestimated the interactions in a region beyond the minimum. On the other hand, the 14-7, generalized (4 parameter) Buckingham or Morse potentials as well as Tang–Toennies handled the steep repulsive region better. Conversely, upon decreasing the upper threshold, the fit is apparently improved (most notably for LJ12-6), but by doing so, the information on repulsive part of potential is disregarded. Decreasing the lower threshold (adding more low-energy points at large separations) appears to improve the fit, but this is due to the presence of a larger number of near-0 energy points at the tail of the dissociation curve, artificially decreasing the RMSE, while the fitted potentials were almost identical. Therefore, it is important to keep in mind that dissociation curves with different densities/numbers of points in different regions of the curves are not directly comparable in terms of RMSE.

#### 4.2.2 Fitted Potential Parameters

The parameters corresponding to the well-depth (ϵ) and minimum energy position (proportional to σ but depending on potential) are mostly well behaved among potentials, pertaining to their physical significance. However, the γ and, where applicable, δ often do not follow any trend with regards to molecular weight (see Tables S31–S46). Interestingly, the quality of the fit was, in some cases, reliant upon the enforced parameter boundaries. The algorithm (see Methods), in some cases, converged to a suboptimal solution, when the boundaries were set too loosely. Table S26 lists the settings of the parameter boundaries used.

### 4.3 Combination Rules

#### 4.3.1 CCSD(T)/CBS Reference Level

**Figure 1:**
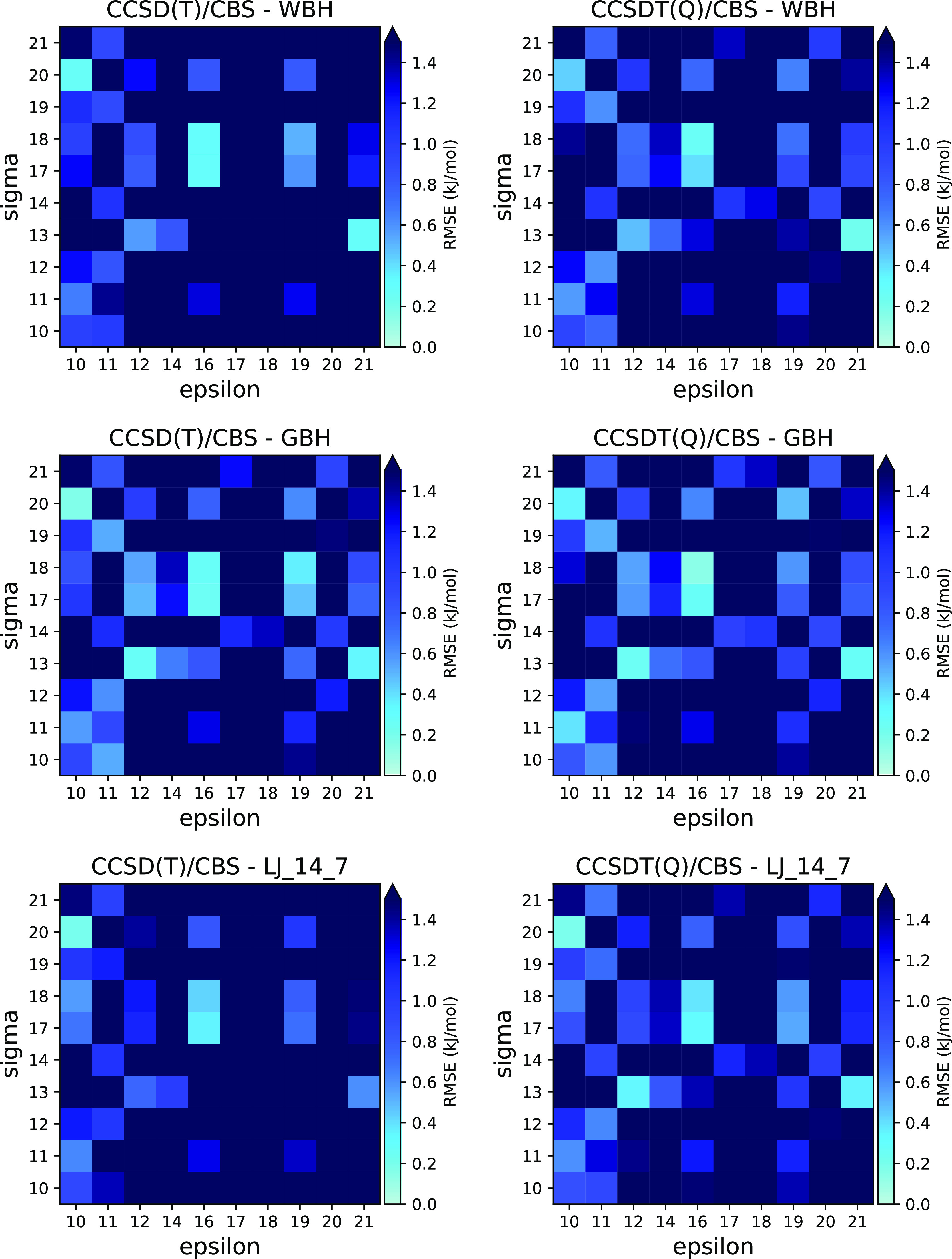
RMSE in the dissociation energy from systematic permutations of combination rules for three potentials and the two highest levels of theory. Similar plots with other potentials are in Figures S3 and S4. Axis labels refer to equations in this paper. For each pair of ϵ and σ, the combination rule with the lowest RMSE was picked among all permutations of the other combination rules.

None of the heterodimer potentials based on combination rules had a RMSE from the reference QM data much less than 0.15 kJ/mol (Table 2), which is a significant drop from the accuracy of the directly fitted potentials (Table 1). Given the less than perfect fit of the homodimers (Table S6), the LJ12-6 and 8-6 potentials also delivered the worst estimate of heterodimer energies by combination rules. The LJ14-7, generalized Buckingham, and Morse potentials were the most accurate, with the best rules featuring the eq 10 for ϵ and eq 20 for σ (Table 3). Following the rules with these two equations, WBH, MBH, MRS, GBH, and LJ14-7 potentials exhibited clusters of combination rules with eqs 16 and 17/18 for ϵ and σ, respectively. eq 21 with 13 for these two parameters in the order given appeared among the best for WBH and MBH, also. Note that the best combination equation for ϵ is always accompanied by one or two specific equations for σ (Table 3, Figure 1). Interestingly, the rule set of eq 16 for ϵ and eq 17 for σ recommended for the 12-6 potential by Waldman and Hagler^16^ did not provide a low RMSE value for this particular potential (due to the combinations of dissimilar heavy-light elements)—while being among the most accurate for other potentials. In contrast, geometric rule 10 was more accurate when applied to both of the parameters of Lennard-Jones 12-6 potential in our fitted models. The new rule eq 20 for σ was the most accurate for six out of nine potentials at the CCSD(T)/CBS level, always accompanied by eq 10 for ϵ (Table S12). When considering individual noble gas pairs, there often are different sets of combination rules that are best for each particular noble gas pair; the best combination rule for He–Ne is different from the best one for the He–Ar pair, with the same potential. Additionally, there are challenging pairs for each potential that are more difficult to describe with any of the combination rule sets used (often He or Ne and heavier noble gas), while for some element pairs (similar weight pairs, like He and Ne), much lower RMSE can be achieved with the “right” combination rule. This can result in a decrease in the RMSE compared to the average for all pairs of an order of magnitude or more.

**Table 2:** RMSE (kJ/mol) from Potentials Based on the Best Combination Rules to Quantum Chemical Data for Different Levels of Theory^a^

level of theory	LJ12-6	LJ8-6	WBH	MBH	MRS	BHA	GBH	LJ14-7	TT
# params	2	2	3	3	3	3	4	4	5
SAPT/TZ	0.802	0.532	0.201	0.197	0.169	0.309	0.218	0.152	0.203
SAPT/QZ	0.889	0.644	0.192	0.225	0.218	0.350	0.267	0.145	0.181
CCSD(T)/TZ	0.911	0.582	0.280	0.271	0.160	0.337	0.182	0.163	0.266
CCSD(T)/QZ	0.976	0.635	0.252	0.278	0.196	0.362	0.215	0.184	0.315
CCSD(T)/CBS	0.978	0.683	0.281	0.274	0.234	0.383	0.177	0.204	0.349
CCSDT(Q)/CBS	0.940	0.612	0.230	0.283	0.236	0.347	0.143	0.188	0.297

a Repulsive energy cutoff of 20 kJ/mol, long range cut at 10% of the well-depth.

**Table 3:** Fitting RMSE and Best Combination Rules on Data Sets with Different Levels of Theory, 20 kJ/mol Threshold

	LJ12-6	LJ8-6	WBH	MBH	MRS	BHA	GBH	LJ14-7	TT
param	ϵ σ	ϵ σ	ϵ σ γ	ϵ σ γ	ϵ σ γ	A *C*_6_ b	ϵ σ γ δ	ϵ σ γ δ	A b *C*_6_ *C*_8_ *C*_10_
CCSD(T)/CBS									
RMSE fit	0.293	0.277	0.090	0.040	0.031	0.040	0.014	0.015	0.011
RMSE comb	0.978	0.683	0.281	0.274	0.234	0.383	0.177	0.204	0.349
rule	10 10	10 20	10 20 21	10 20 14	10 20 14	10 19 19	10 20 14 10	10 20 11 11	19 14 19 10 11
SAPT/QZ									
RMSE fit	0.264	0.254	0.075	0.028	0.036	0.028	0.010	0.011	0.008
RMSE comb	0.889	0.644	0.192	0.225	0.218	0.350	0.267	0.145	0.181
rule	10 10	10 20	21 13 15	21 13 15	10 20 14	10 19 10	21 13 15 20	10 20 17 14	19 14 18 18 10
SAPT/TZ									
RMSE fit	0.259	0.235	0.065	0.023	0.036	0.023	0.009	0.009	0.011
RMSE comb	0.802	0.532	0.201	0.197	0.169	0.309	0.218	0.152	0.203
rule	10 10	16 18	21 13 15	21 13 15	10 20 14	10 19 10	21 13 15 20	10 20 20 19	19 14 18 18 10
CCSD(T)/QZ									
RMSE fit	0.285	0.258	0.079	0.033	0.028	0.033	0.012	0.009	0.007
RMSE comb	0.976	0.635	0.252	0.278	0.196	0.362	0.215	0.184	0.315
rule	10 10	10 20	21 13 15	21 13 15	10 20 14	10 19 19	16 18 14 11	10 20 11 20	10 10 14 10 11
CCSD(T)/TZ									
RMSE fit	0.292	0.241	0.070	0.028	0.027	0.028	0.010	0.008	0.006
RMSE comb	0.911	0.582	0.280	0.271	0.160	0.337	0.182	0.163	0.266
rule	10 10	16 17	16 18 11	16 17 14	16 18 14	10 19 19	16 18 14 11	10 20 11 20	10 10 14 11 11
CCSDT(Q)/CBS									
RMSE fit	0.238	0.255	0.078	0.033	0.025	0.033	0.012	0.007	0.005
RMSE comb	0.940	0.612	0.230	0.283	0.236	0.347	0.143	0.188	0.297
rule	10 10	10 20	21 13 15	21 13 15	16 18 14	10 19 10	16 18 14 17	10 20 20 11	10 10 14 10 11

Equations 16 and 18 for ϵ and σ, respectively, were suggested by Qi et al.^38^ for the 14-7 Lennard-Jones potential. They do not seem to be the most accurate for the 14-7 potential in our work, as they are outperformed by a combination rule with 10, 20 for ϵ, σ, but after these rules, they are among the next best ones (Figure 1). Oliveira and Hünenberger performed a study of combination rules for molecular liquids as well, but using only three different sets for σ and ϵ in a 12-6 potential.^18^ Although they reproduce the findings of Waldman and Hagler when using experimental σ and ϵ for noble gases, they find that the geometric rule eq 10 for both, alternatively geometric for ϵ and arithmetic (eq 11) for σ yielded a better reproduction of properties of organic liquids. For parameters other than ϵ and σ, no clear trends could be discerned in the sense that there were multiple possibilities with a low RMSE.

The Tang–Toennies potential produced less accurate combined potentials than most other potentials (Table 2), although its fit to the QM data is the best (Table 1). This could point to overfitting, but upon including more points to the fit (by changing thresholds), it did not get better. Interestingly, even though the modified Buckingham and generic Buckingham potentials achieved the same fit, the RMSE of the Buckingham potential for combination rules is always slightly higher, perhaps due to the sensitivity of combination rules to the large numbers to which parameters of the Buckingham potential converge to. For this reason, normalization of parameters has been used for Tang–Toennies-like potentials.^20,68^ However, it should be noted that this was only possible due to the combined well-depth and position of the minimum being known beforehand.

Only a small fraction of the combination rules generated by the permutation of combination equations provides reasonable dissociation curves (Figures 1, S2). The Tang–Toennies potential is somewhat of an exception, since none of the combination rules applied to it were worse than ≈4 kJ/mol RMSE, which, however, is still far from acceptable (Figure S2).

#### 4.3.2 Energy Cutoffs and Combination Rules

The combination rules do not seem to scale well with increasing repulsive energy threshold, although the fit is still very good in absolute numbers (Table S1). Using a 100 kJ/mol threshold, the accuracy of combination rules is not much better than 1 kJ/mol compared to 0.2 with a 20 kJ/mol cutoff threshold. The error is mostly due to interactions between dissimilar elements, and the increase in RMSE for higher thresholds is simply caused by inclusion of larger absolute values in the fitting set. The best combination rules often remain stable for a range of energy cutoffs (Table S1), but they often change at high or low upper energy cutoff thresholds. Nevertheless, they remain relatively useful in the sense that the best rule sets for one threshold do not become very poor for another.

The Morse potential is the most consistent, where the best combination rule (Table S1) and also the few next to best rules remain at their places up to a 100 kJ/mol cutoff. Similarly, the best rule for the Buckingham potential is also very consistent and stable for ϵ and σ. These eq 16 for ϵ and 17/18 for σ are also featured by the best combination rules for most of the potentials at the 5 kJ/mol upper energy threshold (Table S1), matching more closely the region around the equilibrium. In general, eq 16 for ϵ does produce combined values closer to the heterodimer fitted parameters than the geometric rule eq 10 (for example compare Tables S33 and S41), although the combination rules featuring eq 10 often produce slightly smaller overall RMSE in other setups. Changing the lower energy threshold did not seem to impact the results.

#### 4.3.3 Levels of Theory

In a similar manner as for CCSD(T)/CBS at 20 kJ/mol cutoff, the best (and closest to best) combination rules for dissociation curves of different QM methods in general are featuring either eq 10 with 20 or eq 16 with 17/18, for ϵ and σ, respectively. For combinations of GBH, MBH, or WBH with some QM methods, eq 21 for ϵ, in conjunction with 12 for σ appeared (see Table 3). The contextual dependence of the accuracy of combination equations is illustrated in Figure 1.

Rules for parameters other than ϵ and σ are more variable and sensitive to the level of theory. With that being said, for the Morse potential, for example, eq 14 is common among the top rules for γ. The Morse and Buckingham potentials are again the most consistent with regard to the best and close-to-best combination rules. Table 4 lists the best rules for potentials tested here, but there are multiple combinations that should be considered for each of the potentials (Figures 1, S3 and S4).

**Table 4:** Recommended Combination Rules (Equation Used for Each Parameter) for Potentials Based on Both the CCSD(T)/CBS and CCSDT(Q)/CBS Levels of Theory^a^

potential	σ	ϵ	γ	δ
LJ12-6	10	10		
LJ8-6	10	20		
	16	17		
	16	18		
WBH	21	13	15	
	10	20	21	
	16	18	17	
MBH	21	13	15	
	10	20	14	
MRS	10	20	14	
	16	18	14	
	16	17	14	
BHA	10	19	10	
	10	19	19	
GBH	16	18	14	11/17
	21	13	15	20/10
	10	20	14	14/10
LJ14-7	10	20	11	11
	16	17	18	21

a TT potential is not applicable to other substances than noble gases in the manner tested here, see Methods.

### 4.4 Second Virial Coefficients

Tables S14–S25 show the deviation of the second virial coefficients calculated from the QM dissociation curves (see Methods) at their respective levels of theory as well as the potentials fitted to these data (and those produced from them by the combination rules), from the experimental values.

**Figure 2:**
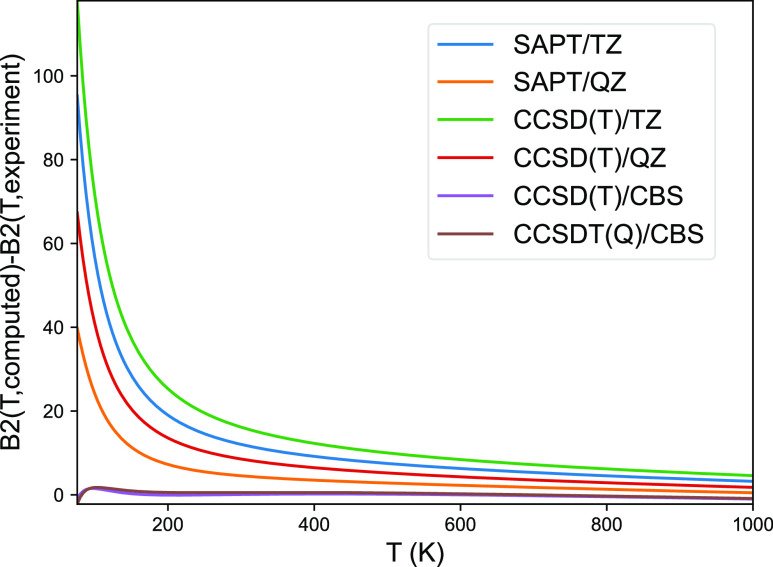
Second virial coefficients for the argon dimer as predicted from quantum chemical dissociation curves relative to the experimental data.

The helium dimer is excluded from the analysis because the quantum nature of helium requires a special treatment (see Methods), but accurate second virial coefficients for helium based on more elaborate computations have been reported elsewhere.^76,77^ For systems other than the helium dimer, there apparently is a progressive improvement of the second virial coefficient estimates with the increasing quality of QM potential, ending with the CCSDT(Q)/CBS that includes relativistic corrections, which provides the most accurate predictions of second virial coefficients (Figure 2). Likewise, Lennard-Jones 14-7 and Tang–Toennies potentials fitted on these QM data also perform well and close to the potentials published earlier^20,60,61,92,96,97^ using the same CCSDT(Q)/CBS reference data (Table S25).

**Figure 3:**
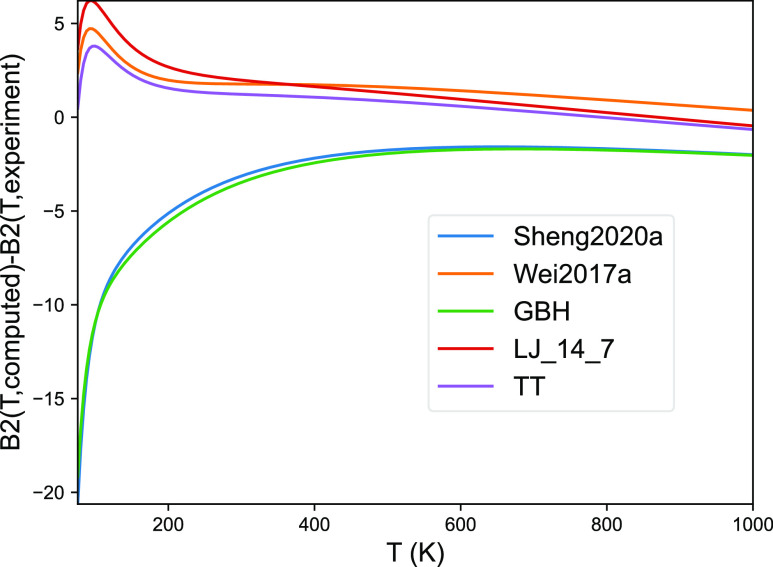
Second virial coefficients for the argon dimer from different models relative to the experimental data. Results based on potentials published by Sheng^68^ and Wei^20^ are given for comparison. The other potentials, GBH, LJ14-7, and TT were derived from the CCSDT(Q) data.

The second virial coefficients from the dissociation curve at CCSD(T)/CBS, the “gold standard”of computational chemistry,^52^ were the second best with very comparable RMSE, but already CCSD(T) in quadruple ζ represents a significant drop in quality compared to the complete basis-set data (Figure 2). These results showcase the sensitivity of the second virial coefficients to the level of theory used. Figure 3 shows comparable numbers for the analytical potentials. Of the analytical potentials, the one proposed by Sheng et al. is somewhat surprisingly less accurate than the simpler potentials. On the other hand, the potential due to Wei et al.^20^ as well as the TT and LJ14-7 potential perform relatively well.

The second virial coefficients from potentials of heterodimers generated by combination rules can be compared with those from directly fitted potentials. For the two highest levels of theory, the deviation from experiment for fitted potentials are given in Tables S22 and S24, respectively, and the corresponding tables for combined potentials are Tables S23 and S25. The use of combination rules yield about 70% higher RMSE for TT, over 200% higher for LJ14-7 while for the MRS potential, the combined results are on par with the fitted results. For GBH, the change in RMSE is positive at the CCSD(T)/CBS level but negative at the CCSDT(Q)/CBS level of theory. Taken together, this suggests that there may be some kind of error compensation related to both shapes of potentials and combination rules applied.

A somewhat puzzling finding was that the second virial coefficient estimates of potentials fitted on the SAPT dissociation curves (triple or quadruple ζ) were more accurate than the coefficients obtained by a direct numerical integration through these data points (Table S14). Closer scrutiny revealed that for some element pairs, the dissociation curve tail (very long distance of about 20 to 30 Å) was ill-behaved for SAPT (they were not converging to 0 rapidly enough or even became positive). The calculation of second virial coefficients is, however, sensitive to values at larger separations due to integration over the volume. Therefore, potentials trained on the SAPT data within cutoffs exhibited better long-range behavior enforced by their functional form than the fitting data itself. Although small differences in the dissociation energy curve tail are practically irrelevant for noncovalent interactions, they are vital for second virial coefficient determination.

### 4.5 Condensed Phase Simulations

**Figure 4:**
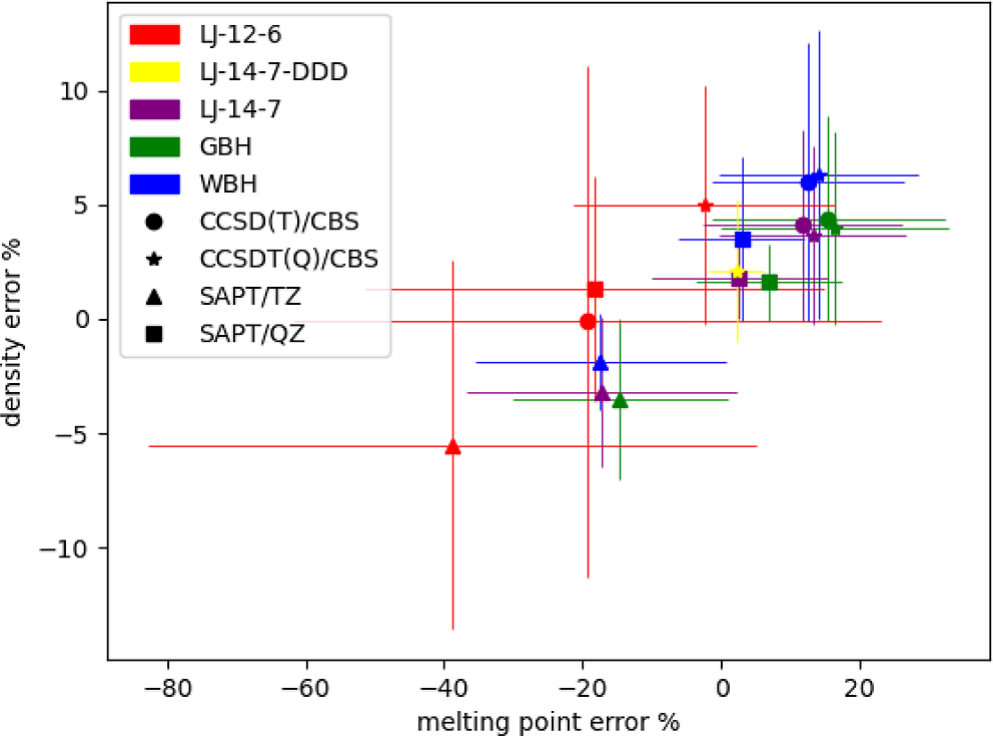
Mean signed error in melting points and densities (at the transition temperature), in percentage of the experimental values for Ne, Ar, Kr, and Xe. Lennard-Jones 12-6 in red, Lennard-Jones 14-7 in purple (yellow with Axilrod–Teller DDD correction), Wang–Buckingham in blue, and generalized Wang–Buckingham in green.

Next, we applied the analytical potentials to molecular simulations of monatomic noble gases in the condensed phase. We tested the commonly used 2-parameter Lennard-Jones 12-6,^4^ 3-parameter Wang–Buckingham,^9^ 4-parameter generalized Buckingham,^45^ as well as the buffered 14-7 potential.^39^ These potentials that represent different mathematical forms (two with exponential function for repulsive interaction, two without) and that span various degrees of effectiveness in fitting the QM potentials were evaluated in molecular dynamics simulations against experimental data: melting points, liquid densities, and enthalpies of vaporization. In what follows, we discuss results for potentials fitted on the CCSD(T)/CBS, CCSDT(Q)/CBS, and SAPT/TZ levels of theory (Figure 4, Tables S27, S2, and S30). In addition, the SAPT/QZ results are given in Table S29. From replicated simulations with the same input, the melting points were found to be reproducible with an uncertainty of 1 K and thus small nonsystematic differences should not be overinterpreted.

In one further set of simulations, the Axilrod–Teller three body potential^35^ was added to the 14-7 potential trained on the CCSDT(Q)/CBS data set. This is a representative case of the van der Waals potential functions capable of reproducing the dissociation energy curve.

#### 4.5.1 CCSD(T)/CBS

All the potentials for CCSD(T)/CBS data are overly attractive, resulting in higher melting points, a behavior that is more pronounced for heavier noble gases (Table S27). Helium is once again a special case. Due to the zero-point energy (ZPE), it does not freeze at atmospheric pressure, while all the potentials except Lennard-Jones 12-6 estimated a melting point of around 8 K. The LJ12-6 predicted the absence of melting point for helium correctly (owing to its ϵ being practically zero, see Table S31). However, it failed completely for neon, for which LJ12-6 also predicted no melting point. Shallowness of the 12-6 potential in general is likely responsible for the lower melting point predictions compared with other potentials.

In general, enthalpies of vaporization and densities at the melting point were overestimated, as well. While decent estimates of melting points and other properties are found for neon and argon, those of krypton and xenon are too high owing to the stronger interatomic interactions.

#### 4.5.2 SAPT/TZ/Triple ζ

SAPT at a triple ζ pertained to shallower well-depths than the other QM methods used for the simulations (Figure S1). This translated into significant differences in melting points compared to the CCSD(T)/CBS results discussed above (see Table S28). In this case, all of melting points were underestimated, while the Lennard-Jones 12-6 with its inherent inadequacy in the potential (as argued in the Analytical Fits section) underestimated them the most. Consistently, enthalpies of vaporization and densities were also lower than for the other QM methods used for training.

#### 4.5.3 CCSDT(Q)/CBS

Due to the corrections in the post-CCSD(T) treatment employed, the potentials of heavier noble gases were actually slightly shallower (Figure S1) than those of CCSD(T)/CBS. Even so, the melting points, densities, and enthalpies of vaporization of krypton and xenon were still overestimated, although a slight improvement over CCSD(T)/CBS is apparent (Table S30). The similarity of CCSDT(Q)/CBS and CCSD(T)/CBS dissociation energy curves (Figure S1) when taken together with the difference in melting points (Table S30) between force fields trained on respective QM-method data set highlights the sensitivity to level of theory of the training data.

The use of the Axilrod–Teller three-body potential leads to a substantial lowering of melting point dependent on the molecular weight (Table 5). These results are discussed below.

**Table 5:** Properties at the Melting Point of Pure Noble Gases in the Liquid Phase and Simulations of the LJ14-7 Force Field Trained with the CCSDT(Q)/CBS at 20 kJ/mol Cutoff Benchmark^a^

element	He	Ne	Ar	Kr	Xe
property					
experiment					
ρ, liquid (g/L)	140.6^b^	1239.6	1416.6	2447.7	2973.8
ρ, solid (g/L)	c	1444.0	1623.0	2825.9	3540.0
Δ_vap_*H* (kJ/mol)	0.095^b^	1.76	6.54	9.19	12.71
melting point (K)	c	24.55	83.78	115.78	161.36
LJ14-7					
ρ, liquid (g/L)	297	1321	1456	2522	3060
ρ, solid (g/L)	344	1538	1676	2897	3494
Δ_vap_*H* (kJ/mol)	0.51	2.13	7.20	10.20	14.37
melting point (K)	7.0	28.0	94.0	132.5	186.5
LJ14-7-DDD					
ρ, liquid (g/L)	291	1315	1432	2472	2991
ρ, solid (g/L)	339	1507	1629	2833	3376
Δ_vap_*H* (kJ/mol)	0.49	2.08	6.79	9.54	13.31
melting point (K)	7.0	26.5	84.5	117.0	160.5

a Experimental values are taken from https://app.knovel.com/kn.^98^ Calculated densities ρ and vaporization enthalpies Δ_vap_*H* are averaged from the last 20% of the respective simulation at the melting point for the models established here. The solid densities were established at 0.5 K below the melting point. The first temperature at which the box was melted is considered the melting point. As the duration of simulation were 2 ns or longer, until a single phase is left in the box, the uncertainty in melting points given by convergence within a same run is also 0.5 K. The uncertainty given independent replica runs is estimated to be 1 K. DDD indicates that three-body dispersion is included according to Axilrod–Teller formula, with the three-body coefficients taken from ref (72). Standard error was estimated from the time correlation of the density and energy time series.^99^ The uncertainty for densities is below 2 g/L, and for enthalpies below 0.02 kJ/mol.

b Experimental properties of liquid He were determined at *T* = 3 K.

c Solid He does not exist at ambient pressure.

#### 4.5.4 Insights from Condensed Phase Simulations

Even though we were able to converge to a good correspondence to the experimental values of second virial coefficients with the increasing level of theory, none of the QM-derived potentials without the three-body correction delivered a satisfying result for the bulk simulations when used for the force field training. Figure 4 summarizes the average deviation from melting temperature and liquid density obtained for Ne, Ar, Kr, and Xe using four potentials fitted on four levels of theory. The SAPT/TZ underestimate both properties, while CCSDT(Q)/CBS overestimates both. It seems that the SAPT/QZ-based potentials (except LJ12-6) are relatively close to zero, but for example, a good result for the combination of SAPT/QZ training data and the generalized Buckingham potential (Table S29) for Ne and Ar is likely an instance of error compensation since the results for Kr and Xe are not as close to experiment for this combination. For noble gases, ZPEs (mostly for helium) as well as many-body dispersion may result in a decrease in the melting points and densities as they both are repulsive. Although it is possible to compute ZPEs for crystals,^100^ it is not straightforward to devise an effective treatment of ZPEs in classical potentials. As judged from the values obtained from the quantum corrections to the second virial coefficients (eqs 26–28), the ZPE is important at low temperature only and only for dimers involving light elements.^92^

The addition of a three-body correction in the form of the dipole–dipole–dipole Axilrod–Teller potential (eq 23) to the Lennard-Jones 14-7 potential trained on the CCSDT(Q)/CBS level of theory leads to a substantial lowering of melting points, providing estimates remarkably close to experiment (Table 5). The results are consistent with the literature in that the inclusion of the many-body dispersion correction is necessary for accurate reproduction of experimental data and, additionally, that the Axilrod–Teller form of three-body dispersion is a good approximation to achieve this goal.^71^ On the other hand, the enthalpies of vaporization are still somewhat too high. The change of density on going from the solid to the liquid phase is somewhat overestimated (except for Xe) without three-body dispersion, while it is somewhat underestimated when the Axilrod–Teller term is taken into account. For Xenon, phase change should be accompanied by a density change of about 566 g/L, whereas our pair potential predicts 434 g/L and the model including three-body dispersion just 385 g/L. The density of the solid phase of Xe is too low, in particular when using the three-body potential, the reason for which is unknown to the authors.

Pahl and co-workers estimated melting points of 86 K for argon and 26 K for neon based on Monte Carlo simulations of clusters using an “extended” Lennard-Jones potential fitted to CCSD(T) with a pentuple zeta basis set, in combination with the Axilrod–Teller three-body dispersion for argon, but neglecting the three body treatment for lighter neon.^101^ When comparing these results to our work, we note that we observe a slightly greater lowering of the melting points due to the explicit Axilrod–Teller correction (from 94 to 84.5 K, Table 5) compared to that from 90.6 to 86.3 K for Ar in the cited work. The discrepancy could be due to the use of short cutoffs by Pahl et al. or the system shape (cluster versus bulk). Those authors also argue that the “vibrational delocalization” (a ZPE related effect) is small for neon (leading to a shift of about 1 K) and was not considered for argon at all.

The best combination rules reproduced the fit within 0.15 kJ/mol combined RMSE on the 20 kJ/mol threshold subset [CCSDT(Q)/CBS, GBH in Table S13]. This may seem as a relatively small price for the reduction of the number of parameters used. However, for He–Xe, for example, the RMSE was 0.246 [CCSD(T)/CBS, GBH; Table S12], which is comparable to the well-depth of the dimer potential itself at this level (0.249 kJ/mol); for Ne–Xe, it amounted to about 35% [CCSD(T)/CBS, TT; Table S12]. Judging by the observed sensitivity of the molecular dynamics simulations to the QM level of theory, such a change in potential well-depth due to a combination rule could cause significant errors in the simulations of these systems. On the other hand, it remains to be determined whether complex combining rules are advantageous for similar-weighted elements, as commonly employed in, e.g., biomolecular simulations.^18,102^

### 4.6 Conclusions

Modeling noble gases is an adventurous task. Their interaction is dispersion-based, and a very high level of theory is needed to reproduce pair potentials relatively accurately. Even so, for the reproduction of (bulk) experimental properties, those QM training data are yet not sufficient, and many-body effects, especially for the heavier ones and potentially ZPE for lighter ones (helium), need to be accounted for. Moreover, for the development of highly accurate potentials, most groups do not even rely on dissociation curves for deriving dispersion parameters but use many-body perturbation theory with time-dependent QM calculations.^95^ This is, however, not practical for compounds other than the simplest compounds such as noble gases. For molecular compounds, quantum chemical dissociation curves at a level of theory that provides a good balance between affordability and accuracy are all that is possible. After extensive benchmarking, different options are available for force field development, such as CCSD(T)/CBS, which, although termed “Gold Standard” of computational chemistry, has its limitations.^103^ SAPT has been hailed as a breakthrough tool for force field development as well^67^ and the SAPT2 + (3)δMP2 level of theory was termed the “Gold Standard” among SAPT methods.^65^ Here, we used the even more accurate SAPT2 + (CCD)δMP2 method,^65^ but as we have shown, the method is quite a bit behind CCSD(T)/CBS in accuracy for reproducing gas-phase interactions. In addition, there seems to be an issue, potentially of numerical origin, at long distances where interaction energies even become positive in some cases. On the bright side, we suspect that this need for a high level of theory will decrease for systems other than noble gases owing to the lower effective importance of dispersion for elements abundant in commonly simulated systems (biological systems), and it may be that for those systems, lower levels of theory are sufficient. Likewise, the expensive three-body potential or many-body correction is less important for lighter and/or not-dispersion-dominated systems.

The 12-6 Lennard-Jones potential (or its 8-6 variant) was not able to accommodate the shape of the QM dissociation curve of noble gases very well in our setup. Rather, it tended toward shallower minima, which then greatly impacts the physical properties (from the molecular simulation or second virial coefficients, in this case). On the other hand, excellent fits to the data were achieved with the 5-parameter Tang–Toennies, followed by a four-parameter 14-7 Lennard-Jones potential and generalized 4-parameter Buckingham potential.

Although a very good fit to the QM data can be achieved, conventional combination rules produce errors that could be too large for some pairs of noble gases considering the sensitivity of the simulations to the change in potential well-depth. Despite this, it is possible to find the most accurate combination rule for each potential. The best combination rules vary slightly with the changing level of theory or the energy cutoffs, but general trends and commonly well-performing rule sets can be established. The ϵ and σ parameters that are associated with potential well-depths and position of the minima, respectively, are well-behaved, and the best combination rules for them are quite consistent throughout different setup and training data (Figure 1). Further work on potential-specific combination rules for molecular compounds could start by evaluating the combination rules listed in Tables 4 or 3.

## Data Availability

Inputs to reproduce the calculations presented here are available from github.^91^

## Supporting Information Available

Dissociation curves; fitting at different energy thresholds; analytical fits; combination rules; second viral coefficient; condensed phase simulation results; and force field parameters (PDF)
