# Correction to “Impact of Combination Rules, Level of Theory, and Potential Function on the Modeling of Gas- and Condensed-Phase Properties of Noble Gases”

**DOI:** 10.1021/acs.jctc.5c01981

**Published:** 2025-12-20

**Authors:** Kristian Kříž, Paul J. van Maaren, David van der Spoel

In our recent paper about combination rules,^1^ an error was introduced when summarizing the results in Table 4. Columns labeled σ and ϵ should be swapped, and the correct version is given in Table 4.

**Table 4:** Recommended Combination Rules (Equation Used for Each Parameter) For Potentials Based on Both the CCSD­(T)/CBS and CCSDT­(Q)/CBS Levels of Theory

Potential	σ	ϵ	γ	δ
LJ12-6	10	10		
LJ8-6	20	10		
	17	16		
	18	16		
WBH	13	21	15	
	20	10	21	
	18	16	17	
MBH	13	21	15	
	20	10	14	
MRS	20	10	14	
	18	16	14	
	17	16	14	
BHA	19	10	10	
	19	10	19	
GBH	18	16	14	11/17
	13	21	15	20/10
	20	10	14	14/10
LJ14-7	20	10	11	11
	17	16	18	21
