# Supplementary material for: Impact of Combination Rules, Level of Theory, and Potential Function on the Modeling of Gas- and Condensed-Phase Properties of Noble Gases
Source: J Chem Theory Comput. 2024 Mar 13;20(6):2362–76. doi: 10.1021/acs.jctc.3c01257 (PMC10976648; doi:10.1021/acs.jctc.3c01257)
Supplement: Supplementary file 1 — ct3c01257_si_001.pdf [file ct3c01257_si_001.pdf]

Impact of Combination Rules, Level of Theory and Potential Function on the Modelling of Gas and Condensed Phase Properties of Noble Gases - Supplementary Information

Kristian Kříž, Paul J. van Maaren, and David van der Spoel*

Department of Cell and Molecular Biology, Uppsala University, Box 596, SE-75124 Uppsala, Sweden

E-mail: david.vanderspoel@icm.uu.se

Phone: +46 18 471 4205

Contents

1 Dissociation Curves **S2**

2 Fitting at Different Energy Thresholds **S4**

3 Analytical Fits **S6**

4 Combination Rules **S10**

5 Second Virial Coefficient **S16**

6 Condensed Phase Simulation Results **S24**

7 Force Field Parameters **S28**

1 Dissociation Curves

Figure S1: Dimer energies (kJ/mol) as a function of distance (Å) at different levels of theory.

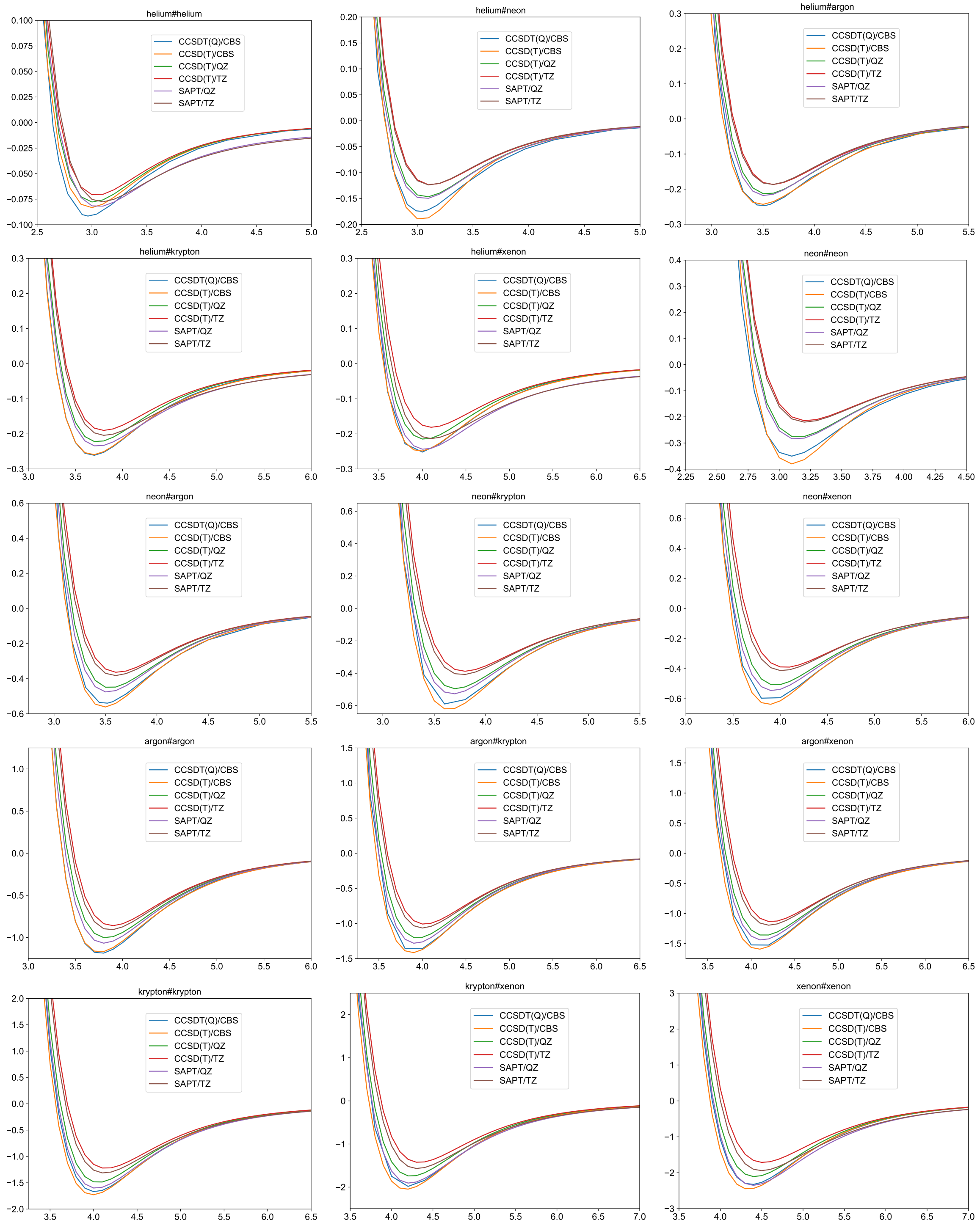

2 Fitting at Different Energy Thresholds

Table S1: Fitting RMSE and best combination rules on datasets with different upper energy thresholds. Level of theory used CCSD(T)/CBS.

	LJ12-6	LJ8-6	WBH	MBH	MRS	BHA	GBH	LJ14-7	TT
Param.	ϵ σ	ϵ σ	ϵ σ γ	ϵ σ γ	ϵ σ γ	A b C_6	ϵ σ γ δ	ϵ σ γ δ	A b C_6 C_8 C_{10}
Treshold 5 kJ/mol									
RMSE fit	0.056	0.195	0.061	0.023	0.027	0.023	0.012	0.007	0.005
RMSE comb	0.195	0.216	0.104	0.097	0.089	0.148	0.094	0.096	0.059
Rule	16 18	16 17	16 18 18	16 18 17	16 20 17	10 10 14	16 18 15 14	16 18 20 12	10 19 10 14 11
Treshold 10 kJ/mol									
RMSE fit	0.103	0.236	0.072	0.030	0.029	0.030	0.012	0.008	0.007
RMSE comb	0.480	0.306	0.146	0.158	0.136	0.210	0.148	0.153	0.137
Rule	10 10	10 20	16 18 17	16 17 17	16 20 18	10 10 14	10 20 15 17	10 20 14 20	10 19 11 11 11
Treshold 15 kJ/mol									
RMSE fit	0.176	0.262	0.082	0.035	0.030	0.035	0.013	0.011	0.009
RMSE comb	0.671	0.416	0.188	0.190	0.192	0.287	0.166	0.191	0.216
Rule	10 10	10 20	21 13 15	21 13 15	10 20 21	10 19 10	10 20 14 11	10 20 11 11	10 19 11 11 11
Treshold 20 kJ/mol									
RMSE fit	0.293	0.277	0.090	0.040	0.031	0.040	0.014	0.015	0.011
RMSE comb	0.978	0.683	0.281	0.274	0.234	0.383	0.177	0.204	0.347
Rule	10 10	10 20	10 20 21	10 20 14	10 20 14	10 19 19	10 20 14 10	10 20 11 11	19 14 19 10 11
Treshold 30 kJ/mol									
RMSE fit	0.539	0.308	0.106	0.050	0.031	0.050	0.018	0.017	0.014
RMSE comb	1.574	1.156	0.411	0.404	0.275	0.521	0.278	0.277	0.508
Rule	10 10	16 18	10 20 14	10 20 14	10 20 14	10 19 19	10 20 11 19	10 20 11 11	10 11 14 10 11
Treshold 40 kJ/mol									
RMSE fit	0.698	0.322	0.120	0.059	0.032	0.059	0.022	0.023	0.036
RMSE comb	1.771	1.358	0.531	0.584	0.301	0.615	0.406	0.566	0.673
Rule	10 10	16 18	16 17 18	10 20 14	10 20 14	10 19 19	10 20 12 19	10 20 11 11	10 19 14 11 11
Treshold 50 kJ/mol									
RMSE fit	1.041	0.355	0.136	0.070	0.034	0.070	0.027	0.025	0.038
RMSE comb	2.472	2.140	0.494	0.628	0.357	0.749	0.418	0.486	0.887
Rule	10 10	10 10	12 13 14	10 20 14	10 20 14	10 19 10	10 20 10 19	10 11 14 20	10 12 11 11 11
Treshold 100 kJ/mol									
RMSE fit	2.548	0.629	0.198	0.112	0.058	0.283	0.056	0.036	0.042
RMSE comb	5.325	2.680	1.058	1.076	0.774	1.238	0.966	0.976	1.892
Rule	10 10	10 10	12 13 21	12 13 14	16 18 21	10 19 10	10 20 14 19	21 13 14 11	10 12 11 11 11

3 Analytical Fits

Table S2: Root mean square error (kJ/mol) from analytical fit of potentials to quantum chemical data at the SAPT/TZ level of theory. Repulsive energy cut-off 20 kJ/mol, long range cut at 10% of the well-depth.

Pair	LJ12-6	LJ8-6	WBH	MBH	MRS	BHA	GBH	LJ14-7	TT
He-He	0.541	0.117	0.009	0.006	0.011	0.006	0.008	0.013	0.006
He-Ne	0.393	0.074	0.024	0.009	0.008	0.009	0.009	0.013	0.003
He-Ar	0.282	0.079	0.033	0.014	0.009	0.014	0.009	0.012	0.003
He-Kr	0.196	0.059	0.018	0.006	0.020	0.006	0.009	0.020	0.006
He-Xe	0.182	0.056	0.015	0.009	0.024	0.009	0.011	0.007	0.009
Ne-Ne	0.131	0.144	0.024	0.005	0.019	0.005	0.006	0.003	0.003
Ne-Ar	0.208	0.193	0.044	0.014	0.023	0.014	0.008	0.008	0.003
Ne-Kr	0.142	0.183	0.040	0.012	0.023	0.012	0.006	0.006	0.002
Ne-Xe	0.224	0.199	0.049	0.017	0.022	0.017	0.010	0.009	0.004
Ar-Ar	0.177	0.288	0.080	0.031	0.032	0.031	0.008	0.005	0.007
Ar-Kr	0.211	0.318	0.093	0.037	0.034	0.037	0.011	0.006	0.007
Ar-Xe	0.186	0.344	0.101	0.042	0.035	0.042	0.012	0.004	0.008
Kr-Kr	0.254	0.331	0.095	0.033	0.052	0.033	0.009	0.007	0.011
Kr-Xe	0.230	0.350	0.099	0.032	0.062	0.032	0.009	0.007	0.032
Xe-Xe	0.225	0.362	0.102	0.028	0.078	0.028	0.010	0.007	0.013
RMSE	0.259	0.235	0.065	0.023	0.036	0.023	0.009	0.009	0.011

Table S3: Root mean square error (kJ/mol) from analytical fit of potentials to quantum chemical data at the SAPT/QZ level of theory. Repulsive energy cut-off 20 kJ/mol, long range cut at 10% of the well-depth.

Pair	LJ12-6	LJ8-6	WBH	MBH	MRS	BHA	GBH	LJ14-7	TT
He-He	0.543	0.116	0.010	0.005	0.011	0.005	0.007	0.013	0.005
He-Ne	0.355	0.071	0.024	0.008	0.009	0.008	0.007	0.014	0.002
He-Ar	0.270	0.081	0.034	0.014	0.010	0.014	0.009	0.013	0.004
He-Kr	0.304	0.073	0.028	0.009	0.019	0.009	0.012	0.020	0.008
He-Xe	0.276	0.068	0.025	0.010	0.023	0.010	0.013	0.023	0.010
Ne-Ne	0.138	0.142	0.030	0.009	0.017	0.009	0.004	0.007	0.001
Ne-Ar	0.201	0.202	0.051	0.018	0.023	0.018	0.008	0.008	0.003
Ne-Kr	0.245	0.214	0.059	0.023	0.023	0.023	0.010	0.008	0.006
Ne-Xe	0.222	0.212	0.060	0.025	0.022	0.025	0.010	0.007	0.006
Ar-Ar	0.153	0.296	0.080	0.028	0.039	0.028	0.008	0.005	0.004
Ar-Kr	0.189	0.337	0.100	0.040	0.039	0.040	0.012	0.004	0.007
Ar-Xe	0.168	0.361	0.110	0.047	0.037	0.047	0.015	0.005	0.008
Kr-Kr	0.227	0.364	0.112	0.043	0.053	0.043	0.009	0.005	0.013
Kr-Xe	0.203	0.391	0.118	0.044	0.061	0.044	0.007	0.007	0.015
Xe-Xe	0.186	0.401	0.123	0.041	0.076	0.041	0.006	0.008	0.015
RMSE	0.264	0.254	0.075	0.028	0.036	0.028	0.010	0.011	0.008

Table S4: Root mean square error (kJ/mol) from analytical fit of potentials to quantum chemical data at the CCSDT/TZ level of theory. Repulsive energy cut-off 20 kJ/mol, long range cut at 10% of the well-depth.

Pair	LJ12-6	LJ8-6	WBH	MBH	MRS	BHA	GBH	LJ14-7	TT
He-He	0.712	0.156	0.023	0.010	0.006	0.010	0.008	0.008	0.004
He-Ne	0.405	0.076	0.024	0.009	0.008	0.009	0.009	0.013	0.003
He-Ar	0.293	0.077	0.032	0.014	0.009	0.014	0.010	0.012	0.004
He-Kr	0.213	0.076	0.028	0.011	0.010	0.011	0.008	0.011	0.003
He-Xe	0.209	0.081	0.029	0.012	0.009	0.012	0.008	0.008	0.004
Ne-Ne	0.134	0.144	0.025	0.006	0.019	0.006	0.007	0.003	0.003
Ne-Ar	0.216	0.190	0.044	0.015	0.021	0.015	0.008	0.008	0.007
Ne-Kr	0.147	0.181	0.038	0.011	0.022	0.011	0.006	0.006	0.002
Ne-Xe	0.236	0.189	0.048	0.017	0.021	0.017	0.010	0.008	0.004
Ar-Ar	0.186	0.281	0.079	0.031	0.030	0.031	0.008	0.006	0.007
Ar-Kr	0.226	0.306	0.089	0.036	0.034	0.036	0.010	0.006	0.007
Ar-Xe	0.201	0.330	0.097	0.040	0.036	0.040	0.011	0.004	0.009
Kr-Kr	0.265	0.336	0.103	0.042	0.039	0.042	0.011	0.008	0.008
Kr-Xe	0.234	0.368	0.114	0.047	0.041	0.047	0.013	0.006	0.010
Xe-Xe	0.208	0.407	0.126	0.053	0.045	0.053	0.015	0.005	0.010
RMSE	0.292	0.241	0.070	0.028	0.027	0.028	0.010	0.008	0.006

Table S5: Root mean square error (kJ/mol) from analytical fit of potentials to quantum chemical data at the CCSDT/QZ level of theory. Repulsive energy cut-off 20 kJ/mol, long range cut at 10% of the well-depth.

Pair	LJ12-6	LJ8-6	WBH	MBH	MRS	BHA	GBH	LJ14-7	TT
He-He	0.672	0.152	0.022	0.010	0.006	0.010	0.010	0.008	0.004
He-Ne	0.377	0.075	0.025	0.009	0.008	0.009	0.008	0.014	0.002
He-Ar	0.276	0.079	0.033	0.013	0.010	0.013	0.009	0.013	0.004
He-Kr	0.208	0.082	0.031	0.013	0.010	0.013	0.008	0.010	0.003
He-Xe	0.311	0.091	0.040	0.019	0.010	0.019	0.012	0.010	0.006
Ne-Ne	0.143	0.140	0.031	0.009	0.017	0.009	0.004	0.007	0.001
Ne-Ar	0.210	0.198	0.050	0.018	0.022	0.018	0.008	0.008	0.003
Ne-Kr	0.257	0.208	0.060	0.023	0.021	0.023	0.010	0.009	0.005
Ne-Xe	0.232	0.207	0.061	0.026	0.020	0.026	0.010	0.007	0.006
Ar-Ar	0.160	0.292	0.079	0.028	0.036	0.028	0.008	0.005	0.004
Ar-Kr	0.194	0.328	0.096	0.038	0.038	0.038	0.011	0.005	0.007
Ar-Xe	0.179	0.339	0.105	0.044	0.038	0.044	0.013	0.004	0.014
Kr-Kr	0.228	0.373	0.117	0.050	0.041	0.050	0.014	0.005	0.010
Kr-Xe	0.207	0.397	0.130	0.057	0.042	0.057	0.017	0.005	0.011
Xe-Xe	0.179	0.445	0.145	0.065	0.046	0.065	0.023	0.011	0.013
RMSE	0.285	0.258	0.079	0.033	0.028	0.033	0.012	0.009	0.007

Table S6: Root mean square error (kJ/mol) from analytical fit of potentials to quantum chemical data at the CCSD(T)/CBS level of theory. Repulsive energy cut-off 20 kJ/mol, long range cut at 10% of the well-depth.

Pair	LJ12-6	LJ8-6	WBH	MBH	MRS	BHA	GBH	LJ14-7	TT
He-He	0.645	0.148	0.023	0.010	0.007	0.010	0.010	0.008	0.004
He-Ne	0.351	0.068	0.023	0.009	0.015	0.009	0.007	0.025	0.008
He-Ar	0.268	0.085	0.036	0.016	0.013	0.016	0.011	0.012	0.006
He-Kr	0.327	0.090	0.041	0.018	0.012	0.018	0.012	0.013	0.006
He-Xe	0.303	0.093	0.042	0.020	0.011	0.020	0.012	0.010	0.006
Ne-Ne	0.265	0.147	0.038	0.022	0.035	0.022	0.017	0.032	0.022
Ne-Ar	0.213	0.204	0.057	0.021	0.021	0.021	0.005	0.014	0.003
Ne-Kr	0.260	0.212	0.067	0.028	0.020	0.028	0.007	0.016	0.004
Ne-Xe	0.234	0.218	0.072	0.033	0.017	0.033	0.008	0.010	0.005
Ar-Ar	0.142	0.308	0.082	0.028	0.043	0.028	0.009	0.002	0.008
Ar-Kr	0.178	0.345	0.101	0.039	0.044	0.039	0.010	0.003	0.010
Ar-Xe	0.159	0.361	0.110	0.045	0.043	0.045	0.014	0.002	0.009
Kr-Kr	0.212	0.398	0.130	0.058	0.040	0.058	0.018	0.006	0.012
Kr-Xe	0.188	0.425	0.140	0.062	0.046	0.062	0.019	0.008	0.014
Xe-Xe	0.288	0.515	0.187	0.092	0.043	0.092	0.032	0.021	0.020
RMSE	0.293	0.277	0.090	0.040	0.031	0.040	0.014	0.015	0.011

Table S7: Root mean square error (kJ/mol) from analytical fit of potentials to quantum chemical data at the CCSDT(Q)/CBS level of theory. Repulsive energy cut-off 20 kJ/mol, long range cut at 10% of the well-depth.

Pair	LJ12-6	LJ8-6	WBH	MBH	MRS	BHA	GBH	LJ14-7	TT
He-He	0.637	0.151	0.024	0.011	0.006	0.011	0.010	0.008	0.003
He-Ne	0.039	0.048	0.011	0.003	0.006	0.003	0.001	0.001	0.000
He-Ar	0.099	0.076	0.020	0.007	0.007	0.007	0.002	0.003	0.001
He-Kr	0.211	0.091	0.033	0.014	0.008	0.014	0.007	0.008	0.003
He-Xe	0.320	0.093	0.043	0.020	0.010	0.020	0.011	0.010	0.005
Ne-Ne	0.135	0.155	0.032	0.009	0.017	0.009	0.003	0.005	0.000
Ne-Ar	0.030	0.134	0.029	0.009	0.015	0.009	0.002	0.001	0.000
Ne-Kr	0.255	0.219	0.063	0.025	0.023	0.025	0.008	0.009	0.003
Ne-Xe	0.229	0.220	0.065	0.027	0.021	0.027	0.009	0.008	0.004
Ar-Ar	0.090	0.264	0.073	0.028	0.027	0.028	0.008	0.003	0.003
Ar-Kr	0.196	0.347	0.104	0.044	0.036	0.044	0.013	0.005	0.007
Ar-Xe	0.175	0.371	0.113	0.049	0.036	0.049	0.016	0.006	0.007
Kr-Kr	0.142	0.341	0.102	0.042	0.033	0.042	0.013	0.005	0.006
Kr-Xe	0.119	0.396	0.122	0.052	0.039	0.052	0.021	0.010	0.008
Xe-Xe	0.201	0.437	0.149	0.067	0.039	0.067	0.024	0.011	0.011
RMSE	0.238	0.255	0.078	0.033	0.025	0.033	0.012	0.007	0.005

4 Combination Rules

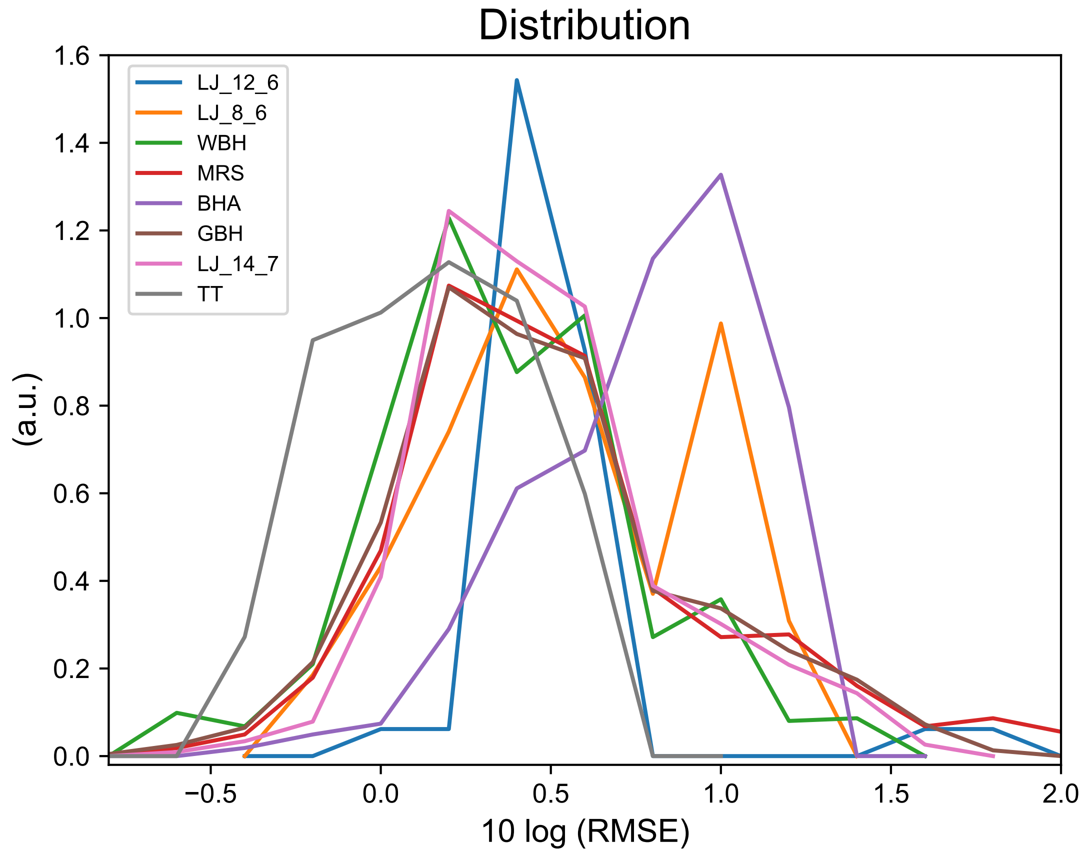

Figure S2: Distribution of root mean square errors from dissociation curves of heterodimers at the CCSD(T)/CBS level of theory, when computed using all possible different permutations of combination relations for parameters.

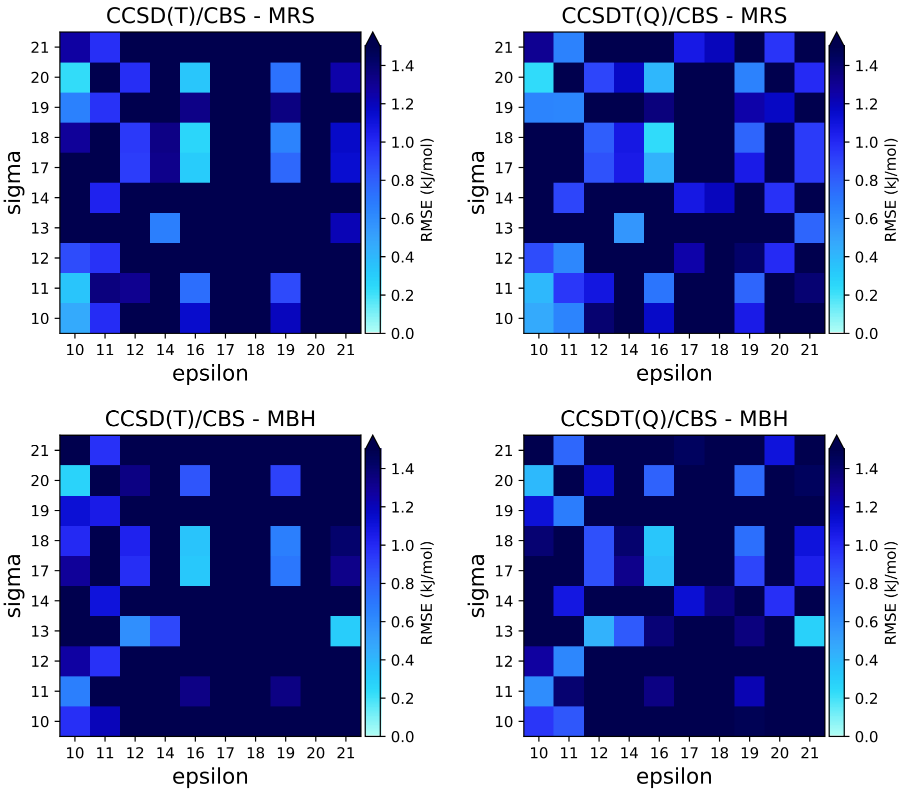

Figure S3: Root mean square error (RMSE) in the dissociation energy from systematic permutations of combination rules for the MRS and MBH potentials and the two highest levels of theory. Axis labels refer to equations in this paper. For each pair of ϵ and σ the combination rule with the lowest RMSE was picked among all permutations of the other combination rules.

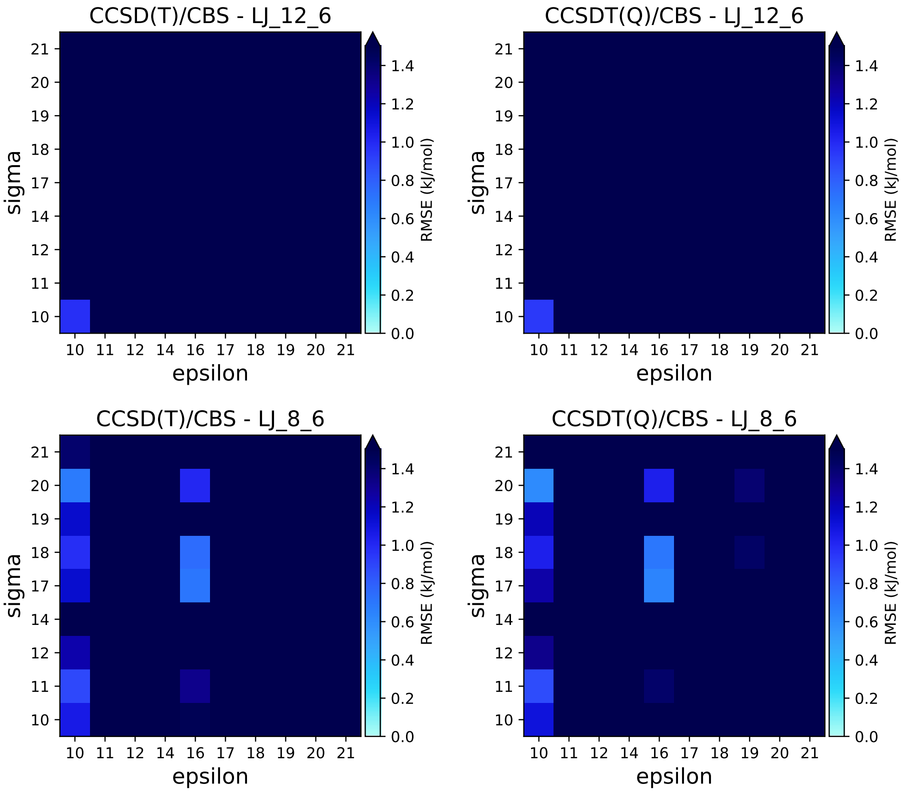

Figure S4: Root mean square error (RMSE) in the dissociation energy from systematic permutations of combination rules for two Lennard-Jones potentials and the two highest levels of theory. Axis labels refer to equations in this paper. For each pair of ϵ and σ the combination rule with the lowest RMSE was picked among all permutations of the other combination rules.

Table S8: Root mean square error (kJ/mol) from of combination-rule based interaction energies from heterodimer potentials at the SAPT/TZ level of theory. Repulsive energy cut-off 20 kJ/mol, long range cut at 10% of the well-depth.

Pair	LJ12-6	LJ8-6	WBH	MBH	MRS	BHA	GBH	LJ14-7	TT
Eq.	ϵ:10<br>σ:10	ϵ:16<br>σ:18	ϵ:21<br>σ:13<br>γ:15	ϵ:21<br>σ:13<br>γ:15	ϵ:10<br>σ:20<br>γ:14	A:10<br>C_6:19<br>b:10	ϵ:21<br>σ:13<br>γ:15<br>δ:20	ϵ:10<br>σ:20<br>γ:20<br>δ:19	A:19<br>b:14<br>C_6:18<br>C_8:18<br>C_{10}:10
He-Ne	0.597	0.432	0.305	0.275	0.350	0.632	0.357	0.084	0.373
He-Ar	0.949	0.800	0.259	0.302	0.072	0.140	0.290	0.063	0.399
He-Kr	1.077	0.780	0.279	0.269	0.186	0.246	0.288	0.121	0.126
He-Xe	1.267	0.958	0.315	0.312	0.241	0.489	0.368	0.183	0.194
Ne-Ar	0.395	0.203	0.085	0.096	0.049	0.164	0.111	0.070	0.175
Ne-Kr	0.706	0.184	0.100	0.103	0.100	0.205	0.109	0.102	0.050
Ne-Xe	1.229	0.226	0.099	0.132	0.178	0.231	0.116	0.295	0.128
Ar-Kr	0.226	0.318	0.098	0.049	0.044	0.065	0.032	0.067	0.029
Ar-Xe	0.367	0.346	0.134	0.096	0.100	0.290	0.075	0.238	0.071
Kr-Xe	0.241	0.357	0.101	0.040	0.090	0.156	0.023	0.096	0.067
RMSE	0.802	0.532	0.201	0.197	0.169	0.309	0.218	0.152	0.203

Table S9: Root mean square error (kJ/mol) from of combination-rule based interaction energies from heterodimer potentials at the SAPT/QZ level of theory. Repulsive energy cut-off 20 kJ/mol, long range cut at 10% of the well-depth.

Pair	LJ12-6	LJ8-6	WBH	MBH	MRS	BHA	GBH	LJ14-7	TT
Eq.	ϵ:10<br>σ:10	ϵ:10<br>σ:20	ϵ:21<br>σ:13<br>γ:15	ϵ:21<br>σ:13<br>γ:15	ϵ:10<br>σ:20<br>γ:14	A:10<br>C_6:19<br>b:10	ϵ:21<br>σ:13<br>γ:15<br>δ:20	ϵ:10<br>σ:20<br>γ:17<br>δ:14	A:19<br>b:14<br>C_6:18<br>C_8:18<br>C_{10}:10
He-Ne	0.623	0.492	0.313	0.276	0.342	0.607	0.329	0.145	0.349
He-Ar	0.822	0.736	0.187	0.266	0.094	0.189	0.277	0.093	0.120
He-Kr	1.294	1.033	0.282	0.364	0.305	0.293	0.424	0.153	0.173
He-Xe	1.629	1.168	0.260	0.394	0.399	0.643	0.514	0.234	0.370
Ne-Ar	0.400	0.234	0.107	0.128	0.068	0.178	0.142	0.079	0.055
Ne-Kr	0.816	0.334	0.126	0.158	0.099	0.249	0.189	0.191	0.093
Ne-Xe	1.213	0.597	0.144	0.138	0.247	0.293	0.150	0.165	0.075
Ar-Kr	0.203	0.337	0.102	0.046	0.045	0.082	0.028	0.029	0.017
Ar-Xe	0.363	0.373	0.124	0.080	0.104	0.323	0.078	0.139	0.057
Kr-Xe	0.216	0.400	0.120	0.050	0.092	0.173	0.024	0.114	0.070
RMSE	0.889	0.644	0.192	0.225	0.218	0.350	0.267	0.145	0.181

Table S10: Root mean square error (kJ/mol) from of combination-rule based interaction energies from heterodimer potentials at the CCSDT/TZ level of theory. Repulsive energy cut-off 20 kJ/mol, long range cut at 10% of the well-depth.

Pair	LJ12-6	LJ8-6	WBH	MBH	MRS	BHA	GBH	LJ14-7	TT
Eq.	ϵ:10<br>σ:10	ϵ:16<br>σ:17	ϵ:16<br>σ:18<br>γ:11	ϵ:16<br>σ:17<br>γ:14	ϵ:16<br>σ:18<br>γ:14	A:10<br>C_6:19<br>b:19	ϵ:16<br>σ:18<br>γ:14<br>δ:11	ϵ:10<br>σ:20<br>γ:11<br>δ:20	A:10<br>b:10<br>C_6:14<br>C_8:10<br>C_{10}:11
He-Ne	0.584	0.455	0.404	0.249	0.326	0.610	0.518	0.303	0.396
He-Ar	0.999	0.789	0.255	0.175	0.054	0.145	0.134	0.067	0.189
He-Kr	1.275	0.850	0.448	0.313	0.212	0.212	0.068	0.168	0.366
He-Xe	1.593	1.081	0.509	0.412	0.218	0.496	0.122	0.198	0.554
Ne-Ar	0.432	0.237	0.059	0.180	0.067	0.303	0.054	0.196	0.091
Ne-Kr	0.770	0.277	0.063	0.163	0.074	0.342	0.083	0.076	0.141
Ne-Xe	1.320	0.407	0.225	0.502	0.142	0.367	0.065	0.213	0.174
Ar-Kr	0.242	0.306	0.090	0.037	0.038	0.072	0.018	0.015	0.012
Ar-Xe	0.382	0.363	0.121	0.209	0.120	0.287	0.085	0.110	0.092
Kr-Xe	0.251	0.381	0.132	0.127	0.098	0.131	0.069	0.031	0.079
RMSE	0.911	0.582	0.280	0.271	0.160	0.337	0.182	0.163	0.266

Table S11: Root mean square error (kJ/mol) from of combination-rule based interaction energies from heterodimer potentials at the CCSDT/QZ level of theory. Repulsive energy cut-off 20 kJ/mol, long range cut at 10% of the well-depth.

Pair	LJ12-6	LJ8-6	WBH	MBH	MRS	BHA	GBH	LJ14-7	TT
Eq.	ϵ:10<br>σ:10	ϵ:10<br>σ:20	ϵ:21<br>σ:13<br>γ:15	ϵ:21<br>σ:13<br>γ:15	ϵ:10<br>σ:20<br>γ:14	A:10<br>C_6:19<br>b:19	ϵ:16<br>σ:18<br>γ:14<br>δ:11	ϵ:10<br>σ:20<br>γ:11<br>δ:20	A:10<br>b:10<br>C_6:14<br>C_8:10<br>C_{10}:11
He-Ne	0.630	0.416	0.276	0.236	0.328	0.586	0.585	0.199	0.407
He-Ar	0.876	0.739	0.234	0.295	0.082	0.187	0.154	0.126	0.157
He-Kr	1.123	0.758	0.261	0.274	0.234	0.189	0.063	0.250	0.334
He-Xe	2.020	1.381	0.517	0.623	0.319	0.624	0.253	0.301	0.797
Ne-Ar	0.448	0.229	0.161	0.177	0.059	0.263	0.026	0.055	0.046
Ne-Kr	0.899	0.312	0.227	0.246	0.110	0.396	0.116	0.171	0.154
Ne-Xe	1.338	0.541	0.200	0.235	0.286	0.400	0.081	0.286	0.108
Ar-Kr	0.210	0.329	0.101	0.050	0.044	0.084	0.025	0.068	0.020
Ar-Xe	0.365	0.352	0.150	0.121	0.085	0.309	0.055	0.073	0.105
Kr-Xe	0.225	0.402	0.133	0.065	0.083	0.145	0.073	0.077	0.088
RMSE	0.976	0.635	0.252	0.278	0.196	0.362	0.215	0.184	0.315

Table S12: Root mean square error (kJ/mol) from of combination-rule based interaction energies from heterodimer potentials at the CCSD(T)/CBS level of theory. Repulsive energy cut-off 20 kJ/mol, long range cut at 10% of the well-depth.

Pair	LJ12-6	LJ8-6	WBH	MBH	MRS	BHA	GBH	LJ14-7	TT
Eq.	ϵ:10 σ:10	ϵ:10 σ:20	ϵ:10 σ:20 γ:21	ϵ:10 σ:20 γ:14	ϵ:10 σ:20 γ:14	A:10 C_6:19 b:19	ϵ:10 σ:20 γ:14 δ:10	ϵ:10 σ:20 γ:11 δ:11	A:19 b:14 C_6:19 C_8:10 C_{10}:11
He-Ne	0.425	0.502	0.333	0.333	0.363	0.620	0.066	0.202	0.374
He-Ar	0.788	0.743	0.141	0.127	0.113	0.257	0.083	0.150	0.173
He-Kr	1.377	1.114	0.284	0.283	0.329	0.220	0.137	0.293	0.523
He-Xe	1.941	1.277	0.380	0.362	0.432	0.565	0.246	0.317	0.813
Ne-Ar	0.446	0.245	0.183	0.167	0.069	0.270	0.133	0.058	0.146
Ne-Kr	0.870	0.320	0.288	0.274	0.119	0.401	0.180	0.104	0.163
Ne-Xe	1.349	0.605	0.518	0.545	0.275	0.500	0.406	0.371	0.221
Ar-Kr	0.199	0.346	0.104	0.045	0.053	0.085	0.033	0.010	0.034
Ar-Xe	0.447	0.381	0.126	0.082	0.079	0.366	0.067	0.108	0.039
Kr-Xe	0.294	0.436	0.143	0.064	0.049	0.160	0.028	0.033	0.054
RMSE	0.978	0.683	0.281	0.274	0.234	0.383	0.177	0.204	0.347

Table S13: Root mean square error (kJ/mol) from of combination-rule based interaction energies from heterodimer potentials at the CCSDT(Q)/CBS level of theory. Repulsive energy cut-off 20 kJ/mol, long range cut at 10% of the well-depth.

Pair	LJ12-6	LJ8-6	WBH	MBH	MRS	BHA	GBH	LJ14-7	TT
Eq.	ϵ:10 σ:10	ϵ:10 σ:20	ϵ:21 σ:13 γ:15	ϵ:21 σ:13 γ:15	ϵ:16 σ:18 γ:14	A:10 C_6:19 b:10	ϵ:16 σ:18 γ:14 δ:17	ϵ:10 σ:20 γ:20 δ:11	A:10 b:10 C_6:14 C_8:18 C_{10}:11
He-Ne	0.222	0.192	0.123	0.134	0.059	0.151	0.210	0.005	0.115
He-Ar	0.521	0.413	0.178	0.192	0.140	0.203	0.157	0.163	0.072
He-Kr	0.952	0.750	0.176	0.263	0.226	0.233	0.136	0.216	0.289
He-Xe	2.094	1.326	0.536	0.700	0.365	0.870	0.104	0.233	0.849
Ne-Ar	0.337	0.207	0.106	0.088	0.016	0.163	0.034	0.129	0.029
Ne-Kr	0.836	0.329	0.144	0.251	0.155	0.243	0.057	0.178	0.095
Ne-Xe	1.478	0.760	0.253	0.311	0.517	0.302	0.253	0.366	0.085
Ar-Kr	0.349	0.419	0.186	0.068	0.041	0.136	0.039	0.019	0.050
Ar-Xe	0.277	0.374	0.117	0.088	0.201	0.329	0.155	0.185	0.162
Kr-Xe	0.150	0.417	0.134	0.068	0.131	0.167	0.110	0.092	0.124
RMSE	0.940	0.612	0.230	0.283	0.236	0.347	0.143	0.188	0.297

5 Second Virial Coefficient

The classical second virial coefficient is computed from[1]

$$B_2(T) \;=\; -2\pi \int_0^\infty (e^{-\beta U(r)} - 1) r^2 dr \tag{S1}$$

with β the Boltzmann factor (1/k_BT). Quantum corrections are implemented according to Bich *et al.*[2]

Table S14: Root mean square error from experimental second virial coefficient for quantum chemical reference SAPT/TZ and fitted potentials, in cm^3/mol. Only data points with experimental B(T) $\geq$ -1000 are taken into account. For training the potentials, the repulsive energy was cut-off at 20 kJ/mol and the long range tail cut at 10% of the well-depth. Integration of eq. S1 was performed from zero to 30 Å.

Element	T (K)	qm	Wei2017a	LJ12-6	MRS	WBH	GBH	LJ14-7	TT
He-Ne	15-345	16.59	1.94	24.05	11.02	3.11	5.99	9.94	7.01
He-Ar	100-740	10.09	1.02	13.73	4.82	1.53	2.25	3.87	3.26
He-Kr	50-345	83.75	0.67	24.60	9.34	3.14	2.45	7.16	1.97
He-Xe	120-345	112.95	1.24	22.04	7.69	4.80	1.09	2.40	1.09
Ne-Ne	50-870	4.82	0.42	10.33	8.41	2.07	4.98	5.10	5.29
Ne-Ar	100-470	8.82	2.48	20.71	15.73	2.25	8.75	9.59	9.98
Ne-Kr	100-490	16.24	3.21	19.29	18.74	4.05	10.72	11.96	11.93
Ne-Xe	160-495	17.03	3.94	24.12	17.59	3.51	9.97	11.17	11.78
Ar-Ar	76-1000	18.37	1.77	28.79	30.82	6.35	17.36	21.93	21.47
Ar-Kr	110-690	35.36	3.93	35.66	38.51	6.99	21.17	27.32	26.99
Ar-Xe	173-695	39.58	3.30	28.29	39.02	3.70	18.56	26.63	26.21
Kr-Kr	110-870	41.72	4.28	44.95	43.25	8.65	23.40	27.80	26.95
Kr-Xe	150-740	82.07	3.60	35.27	41.59	4.74	17.15	22.09	13.25
Xe-Xe	165-965	125.17	2.64	29.86	41.01	9.37	14.23	18.24	15.88
RMSE		58.62	2.76	27.26	27.39	5.15	13.37	17.09	15.82

Table S15: Root mean square error from experimental second virial coefficient for quantum chemical reference SAPT/TZ and combined potentials, in cm^3/mol. Only data points with experimental B(T) $\geq$ -1000 are taken into account. For training the potentials, the repulsive energy was cut-off at 20 kJ/mol and the long range tail cut at 10% of the well-depth. Integration of eq. S1 was performed from zero to 30 Å.

Element	T (K)	qm	Wei2017a	LJ12-6	MRS	WBH	GBH	LJ14-7	TT
He-Ne	15-345	16.59	1.94	24.46	10.89	1.38	6.58	8.12	2.12
He-Ar	100-740	10.09	1.02	12.07	2.38	3.51	5.91	1.13	3.61
He-Kr	50-345	83.75	0.67	24.66	1.28	7.30	11.77	1.50	5.70
He-Xe	120-345	112.95	1.24	20.72	4.85	6.36	10.34	7.02	4.80
Ne-Ar	100-470	8.82	2.48	14.24	12.46	7.87	13.21	6.95	12.61
Ne-Kr	100-490	16.24	3.21	14.69	11.10	10.86	16.98	3.21	12.67
Ne-Xe	160-495	17.03	3.94	7.08	4.61	10.36	15.88	5.76	8.36
Ar-Kr	110-690	35.36	3.93	33.54	35.55	7.20	21.06	24.01	22.33
Ar-Xe	173-695	39.58	3.30	19.14	26.38	4.60	19.18	13.30	10.40
Kr-Xe	150-740	82.07	3.60	34.01	38.22	4.40	19.23	18.51	17.53
RMSE		55.07	2.79	22.13	19.67	6.98	14.90	11.43	11.73

Table S16: Root mean square error from experimental second virial coefficient for quantum chemical reference SAPT/QZ and fitted potentials, in cm^3/mol. Only data points with experimental B(T) $\geq$ -1000 are taken into account. For training the potentials, the repulsive energy was cut-off at 20 kJ/mol and the long range tail cut at 10% of the well-depth. Integration of eq. S1 was performed from zero to 30 Å.

Element	T (K)	qm	Wei2017a	LJ12-6	MRS	WBH	GBH	LJ14-7	TT
He-Ne	15-345	2.65	1.94	23.86	8.60	7.03	3.08	7.31	3.66
He-Ar	100-740	6.02	1.02	12.48	3.36	3.29	0.91	2.28	1.52
He-Kr	50-345	78.38	0.67	27.75	6.63	7.77	0.36	4.61	0.20
He-Xe	120-345	106.19	1.24	26.32	4.92	9.23	2.67	2.84	2.96
Ne-Ne	50-870	2.59	0.42	8.49	5.88	0.96	2.28	2.66	2.81
Ne-Ar	100-470	4.81	2.48	14.58	10.97	4.06	3.36	4.75	5.04
Ne-Kr	100-490	2.51	3.21	19.78	11.99	6.56	3.08	4.88	5.46
Ne-Xe	160-495	2.33	3.94	15.03	10.46	6.94	2.71	4.54	4.89
Ar-Ar	76-1000	7.51	1.77	15.00	19.89	6.28	5.57	9.92	9.02
Ar-Kr	110-690	16.56	3.93	16.23	23.72	10.20	4.84	11.78	11.08
Ar-Xe	173-695	18.33	3.30	7.77	23.05	16.22	2.66	9.65	9.62
Kr-Kr	110-870	58.23	4.28	18.23	22.10	17.44	2.85	7.27	6.09
Kr-Xe	150-740	101.09	3.60	5.24	17.63	28.90	9.53	1.33	3.19
Xe-Xe	165-965	147.99	2.64	10.82	11.90	38.23	18.18	10.30	13.23
RMSE		61.92	2.76	17.14	14.62	15.40	6.22	6.83	6.71

Table S17: Root mean square error from experimental second virial coefficient for quantum chemical reference SAPT/QZ and combined potentials, in cm^3/mol. Only data points with experimental B(T) $\geq$ -1000 are taken into account. For training the potentials, the repulsive energy was cut-off at 20 kJ/mol and the long range tail cut at 10% of the well-depth. Integration of eq. S1 was performed from zero to 30 Å.

Element	T (K)	qm	Wei2017a	LJ12-6	MRS	WBH	GBH	LJ14-7	TT
He-Ne	15-345	2.65	1.94	24.34	8.59	1.17	5.62	6.22	0.84
He-Ar	100-740	6.02	1.02	11.85	1.07	2.90	5.19	1.13	1.00
He-Kr	50-345	78.38	0.67	24.45	2.22	6.33	10.99	5.00	1.27
He-Xe	120-345	106.19	1.24	20.40	8.66	5.47	9.63	10.84	1.54
Ne-Ar	100-470	4.81	2.48	9.18	7.09	2.71	8.04	1.67	6.21
Ne-Kr	100-490	2.51	3.21	7.55	3.88	4.76	10.96	5.01	6.38
Ne-Xe	160-495	2.33	3.94	2.60	5.22	4.71	10.04	13.34	4.14
Ar-Kr	110-690	16.56	3.93	14.12	20.85	8.43	6.25	7.65	8.38
Ar-Xe	173-695	18.33	3.30	4.70	9.79	11.58	4.46	6.85	3.64
Kr-Xe	150-740	101.09	3.60	3.83	13.63	25.74	6.92	4.92	5.88
RMSE		53.22	2.79	14.60	9.82	10.00	8.15	7.20	4.69

Table S18: Root mean square error from experimental second virial coefficient for quantum chemical reference CCSDT/TZ and fitted potentials, in cm^3/mol. Only data points with experimental B(T) $\geq$ -1000 are taken into account. For training the potentials, the repulsive energy was cut-off at 20 kJ/mol and the long range tail cut at 10% of the well-depth. Integration of eq. S1 was performed from zero to 30 Å.

Element	T (K)	qm	Wei2017a	LJ12-6	MRS	WBH	GBH	LJ14-7	TT
He-Ne	15-345	5.80	1.94	24.16	10.98	3.39	5.98	9.92	7.03
He-Ar	100-740	2.81	1.02	13.92	4.95	1.54	2.37	4.03	3.33
He-Kr	50-345	5.95	0.67	26.63	10.63	1.19	5.21	8.45	6.87
He-Xe	120-345	6.24	1.24	24.54	10.53	1.32	5.43	5.53	7.20
Ne-Ne	50-870	5.40	0.42	10.76	8.65	2.35	5.28	5.44	5.55
Ne-Ar	100-470	10.78	2.48	22.46	16.75	3.39	9.94	10.78	12.49
Ne-Kr	100-490	13.81	3.21	22.14	19.99	5.85	12.22	13.34	13.38
Ne-Xe	160-495	12.53	3.94	26.03	19.03	4.40	11.45	12.42	13.14
Ar-Ar	76-1000	23.49	1.77	33.05	33.96	9.65	20.87	25.41	25.09
Ar-Kr	110-690	28.41	3.93	41.45	41.90	11.25	25.03	30.74	30.49
Ar-Xe	173-695	26.60	3.30	34.10	42.26	6.74	22.31	29.66	29.24
Kr-Kr	110-870	35.89	4.28	53.17	51.20	16.87	32.12	38.54	38.34
Kr-Xe	150-740	37.68	3.60	47.67	54.88	13.73	31.64	40.36	39.92
Xe-Xe	165-965	45.30	2.64	50.15	64.41	21.26	39.03	49.26	48.41
RMSE		23.05	2.76	33.23	33.77	9.55	20.04	24.94	24.67

Table S19: Root mean square error from experimental second virial coefficient for quantum chemical reference CCSDT/TZ and combined potentials, in cm^3/mol. Only data points with experimental B(T) ≥ -1000 are taken into account. For training the potentials, the repulsive energy was cut-off at 20 kJ/mol and the long range tail cut at 10% of the well-depth. Integration of eq. S1 was performed from zero to 30 Å.

Element	T (K)	qm	Wei2017a	LJ12-6	MRS	WBH	GBH	LJ14-7	TT
He-Ne	15-345	5.80	1.94	24.54	10.71	3.24	8.56	7.29	10.42
He-Ar	100-740	2.81	1.02	12.16	4.50	2.32	3.60	1.56	4.69
He-Kr	50-345	5.95	0.67	24.76	8.87	4.61	6.96	0.35	9.17
He-Xe	120-345	6.24	1.24	20.70	8.64	4.79	7.11	4.92	8.19
Ne-Ar	100-470	10.78	2.48	15.59	16.67	3.95	9.68	6.41	10.98
Ne-Kr	100-490	13.81	3.21	16.63	19.98	4.85	11.56	4.52	12.65
Ne-Xe	160-495	12.53	3.94	9.64	19.92	5.61	11.83	3.14	12.42
Ar-Kr	110-690	28.41	3.93	39.63	41.89	11.06	25.09	29.41	30.62
Ar-Xe	173-695	26.60	3.30	28.99	44.51	9.10	24.79	23.04	30.70
Kr-Xe	150-740	37.68	3.60	47.48	56.80	15.73	33.55	36.97	41.34
RMSE		18.75	2.79	26.59	28.85	7.64	17.10	17.07	20.75

Table S20: Root mean square error from experimental second virial coefficient for quantum chemical reference CCSDT/QZ and fitted potentials, in cm^3/mol. Only data points with experimental B(T) ≥ -1000 are taken into account. For training the potentials, the repulsive energy was cut-off at 20 kJ/mol and the long range tail cut at 10% of the well-depth. Integration of eq. S1 was performed from zero to 30 Å.

Element	T (K)	qm	Wei2017a	LJ12-6	MRS	WBH	GBH	LJ14-7	TT
He-Ne	15-345	3.23	1.94	23.98	8.82	6.82	3.50	7.59	4.19
He-Ar	100-740	1.42	1.02	13.41	3.68	3.01	1.10	2.63	1.75
He-Kr	50-345	3.07	0.67	21.41	7.84	4.81	2.03	5.32	3.83
He-Xe	120-345	3.22	1.24	27.01	7.20	5.77	1.89	5.12	4.60
Ne-Ne	50-870	3.00	0.42	8.58	6.25	0.87	2.63	3.00	3.21
Ne-Ar	100-470	5.97	2.48	16.66	12.20	2.70	4.75	6.07	6.47
Ne-Kr	100-490	6.57	3.21	21.84	13.94	5.09	5.02	6.95	7.69
Ne-Xe	160-495	5.87	3.94	17.44	12.51	5.20	4.42	6.71	7.23
Ar-Ar	76-1000	13.09	1.77	20.41	24.26	3.15	10.29	14.60	13.89
Ar-Kr	110-690	14.47	3.93	23.16	28.70	5.25	10.40	16.84	16.23
Ar-Xe	173-695	11.28	3.30	13.94	27.35	10.86	6.03	14.36	12.93
Kr-Kr	110-870	16.59	4.28	28.39	32.16	8.26	11.47	19.67	19.14
Kr-Xe	150-740	14.37	3.60	18.28	33.03	12.11	7.40	17.60	17.92
Xe-Xe	165-965	18.36	2.64	16.79	38.06	11.93	10.41	21.22	22.31
RMSE		10.29	2.76	20.08	21.51	7.00	6.76	12.27	12.04

Table S21: Root mean square error from experimental second virial coefficient for quantum chemical reference CCSDT/QZ and combined potentials, in cm^3/mol. Only data points with experimental B(T) ≥ -1000 are taken into account. For training the potentials, the repulsive energy was cut-off at 20 kJ/mol and the long range tail cut at 10% of the well-depth. Integration of eq. S1 was performed from zero to 30 Å.

Element	T (K)	qm	Wei2017a	LJ12-6	MRS	WBH	GBH	LJ14-7	TT
He-Ne	15-345	3.23	1.94	24.44	8.34	2.00	7.79	5.10	8.09
He-Ar	100-740	1.42	1.02	11.96	1.14	2.42	2.32	0.92	3.30
He-Kr	50-345	3.07	0.67	24.56	1.96	5.33	3.85	3.82	5.43
He-Xe	120-345	3.22	1.24	20.38	7.08	4.29	3.66	9.11	4.35
Ne-Ar	100-470	5.97	2.48	10.13	8.36	2.98	4.89	1.62	6.92
Ne-Kr	100-490	6.57	3.21	8.94	5.76	5.08	4.98	4.98	7.92
Ne-Xe	160-495	5.87	3.94	2.84	2.44	4.97	5.43	10.66	8.23
Ar-Kr	110-690	14.47	3.93	21.54	27.33	3.43	10.39	14.46	17.16
Ar-Xe	173-695	11.28	3.30	8.28	21.11	4.37	8.38	5.51	16.93
Kr-Xe	150-740	14.37	3.60	17.86	30.64	10.41	9.58	14.78	19.91
RMSE		8.30	2.79	16.74	15.38	5.05	6.65	8.51	11.32

Table S22: Root mean square error from experimental second virial coefficient for quantum chemical reference CCSD(T)/CBS and fitted potentials, in cm^3/mol. Only data points with experimental B(T) ≥ -1000 are taken into account. For training the potentials, the repulsive energy was cut-off at 20 kJ/mol and the long range tail cut at 10% of the well-depth. Integration of eq. S1 was performed from zero to 30 Å.

Element	T (K)	qm	Wei2017a	LJ12-6	MRS	WBH	GBH	LJ14-7	TT
He-Ne	15-345	0.72	1.94	23.81	6.89	9.72	1.43	5.53	0.99
He-Ar	100-740	1.01	1.02	11.74	2.16	4.70	1.38	1.11	0.85
He-Kr	50-345	0.33	0.67	27.23	4.70	10.32	1.71	2.39	0.81
He-Xe	120-345	1.25	1.24	24.49	4.51	9.57	2.08	2.29	1.73
Ne-Ne	50-870	0.37	0.42	11.21	4.16	4.70	0.72	2.01	0.77
Ne-Ar	100-470	1.13	2.48	10.84	7.09	8.97	1.52	1.05	1.62
Ne-Kr	100-490	2.28	3.21	14.62	7.36	13.20	3.63	1.95	2.14
Ne-Xe	160-495	2.47	3.94	10.03	6.51	12.55	4.06	2.38	2.56
Ar-Ar	76-1000	0.48	1.77	5.94	12.06	14.94	3.28	1.41	0.46
Ar-Kr	110-690	1.83	3.93	5.28	13.59	21.63	6.55	0.91	0.94
Ar-Xe	173-695	5.78	3.30	6.23	12.24	28.28	12.02	3.52	4.36
Kr-Kr	110-870	2.21	4.28	8.06	14.53	25.80	8.91	1.72	2.09
Kr-Xe	150-740	10.16	3.60	9.64	10.56	37.00	18.22	6.60	6.06
Xe-Xe	165-965	7.26	2.64	6.84	13.49	40.97	18.94	5.55	1.22
RMSD		3.91	2.76	14.42	9.42	20.62	8.50	3.27	2.43

Table S23: Root mean square error from experimental second virial coefficient for quantum chemical reference CCSD(T)/CBS and combined potentials, in cm^3/mol. Only data points with experimental B(T) ≥ -1000 are taken into account. For training the potentials, the repulsive energy was cut-off at 20 kJ/mol and the long range tail cut at 10% of the well-depth. Integration of eq. S1 was performed from zero to 30 Å.

Element	T (K)	qm	Wei2017a	LJ12-6	MRS	WBH	GBH	LJ14-7	TT
He-Ne	15-345	0.72	1.94	24.01	6.54	11.44	5.14	1.76	6.60
He-Ar	100-740	1.01	1.02	11.85	1.12	8.95	2.84	2.10	1.48
He-Kr	50-345	0.33	0.67	24.39	4.83	23.91	9.24	6.57	2.28
He-Xe	120-345	1.25	1.24	19.78	10.42	30.70	15.27	12.25	1.82
Ne-Ar	100-470	1.13	2.48	13.26	3.55	14.22	6.38	6.81	4.04
Ne-Kr	100-490	2.28	3.21	13.48	3.07	24.62	14.20	14.32	5.37
Ne-Xe	160-495	2.47	3.94	7.53	7.84	31.79	20.79	20.88	6.09
Ar-Kr	110-690	1.83	3.93	4.59	13.31	21.85	6.85	0.81	2.64
Ar-Xe	173-695	5.78	3.30	8.72	5.77	38.42	20.34	11.81	3.45
Kr-Xe	150-740	10.16	3.60	4.45	10.35	39.44	18.95	7.07	1.65
RMSE		3.95	2.79	14.95	7.60	26.56	13.59	10.38	3.98

Table S24: Root mean square error from experimental second virial coefficient for quantum chemical reference CCSDT(Q)/CBS and fitted potentials, in cm^3/mol. Only data points with experimental B(T) ≥ -1000 are taken into account. For training the potentials, the repulsive energy was cut-off at 20 kJ/mol and the long range tail cut at 10% of the well-depth. Integration of eq. S1 was performed from zero to 30 Å.

Element	T (K)	qm	Wei2017a	LJ12-6	MRS	WBH	GBH	LJ14-7	TT
He-Ne	15-345	4.19	1.94	3.08	6.00	6.29	1.08	1.39	1.21
He-Ar	100-740	0.84	1.02	2.09	2.50	4.06	1.09	0.91	0.85
He-Kr	50-345	0.92	0.67	16.20	4.96	8.50	1.45	1.23	0.97
He-Xe	120-345	1.24	1.24	25.99	4.39	9.92	2.22	2.19	1.76
Ne-Ne	50-870	0.66	0.42	4.78	3.62	4.35	0.62	0.27	0.27
Ne-Ar	100-470	2.12	2.48	2.26	7.64	6.31	0.91	2.46	1.72
Ne-Kr	100-490	2.07	3.21	16.38	8.50	11.74	2.61	1.99	2.31
Ne-Xe	160-495	2.34	3.94	11.75	7.44	11.30	3.24	2.36	2.51
Ar-Ar	76-1000	0.63	1.77	3.56	11.80	15.82	4.21	2.03	1.23
Ar-Kr	110-690	1.94	3.93	10.38	15.50	19.46	4.43	3.71	3.12
Ar-Xe	173-695	2.79	3.30	2.81	14.84	25.51	9.28	2.71	2.77
Kr-Kr	110-870	3.36	4.28	4.44	19.27	23.44	5.47	4.79	4.86
Kr-Xe	150-740	2.51	3.60	12.36	15.47	28.88	12.50	1.38	0.93
Xe-Xe	165-965	3.16	2.64	8.51	22.74	36.06	10.61	3.29	5.70
RMSE		2.31	2.76	11.21	11.99	17.96	5.67	2.47	2.63

Table S25: Root mean square error from experimental second virial coefficient for quantum chemical reference CCSDT(Q)/CBS and combined potentials, in cm^3/mol. Only data points with experimental B(T) $\geq$ -1000 are taken into account. For training the potentials, the repulsive energy was cut-off at 20 kJ/mol and the long range tail cut at 10% of the well-depth. Integration of eq. S1 was performed from zero to 30 Å.

Element	T (K)	qm	Wei2017a	LJ12-6	MRS	WBH	GBH	LJ14-7	TT
He-Ne	15-345	4.19	1.94	24.44	5.69	4.59	5.21	2.20	7.24
He-Ar	100-740	0.84	1.02	12.15	1.55	1.59	0.99	2.73	2.24
He-Kr	50-345	0.92	0.67	24.93	3.62	3.46	0.59	8.21	4.19
He-Xe	120-345	1.24	1.24	20.42	3.39	2.33	1.44	13.66	2.89
Ne-Ar	100-470	2.12	2.48	1.42	7.62	3.23	1.25	4.66	2.20
Ne-Kr	100-490	2.07	3.21	2.10	9.80	2.67	2.14	11.22	3.32
Ne-Xe	160-495	2.34	3.94	8.78	10.57	3.47	2.34	17.17	4.73
Ar-Kr	110-690	1.94	3.93	4.58	16.94	17.59	3.89	2.15	3.91
Ar-Xe	173-695	2.79	3.30	16.00	19.05	21.30	6.24	8.82	5.03
Kr-Xe	150-740	2.51	3.60	9.26	22.04	29.94	7.46	1.70	5.79
RMSE		2.30	2.79	14.93	12.09	13.15	3.90	8.91	4.42

Table S26: The parameter bounds used for the fitting.

Property	Min	Max
	LJ-12-6	
ϵ	0	10
σ	2	20
	LJ-8-6	
ϵ	0	10
σ	2	240
	LJ-14-7	
ϵ	0	10
σ	2	20
γ	0	0.8
δ	0	2
	WBH	
ϵ	0	10
σ	2	20
γ	6	40
	MBH	
ϵ	0	10
σ	2	20
γ	6	30
	MRS	
ϵ	0	10
σ	2	20
γ	1	4
	BHA	
A	500	6E6
B	100	3.5E4
C	2	6
	GBH	
ϵ	0	10
σ	2	20
γ	10	50
δ	0	40
	TT	
A	2E5	1E6
B	8	20
C_6	0	5
C_8	0	4
C_{10}	0	8

6 Condensed Phase Simulation Results

Table S27: Properties at the melting point of pure noble gases in the liquid phase, simulations of force fields trained with the CCSD(T)/CBS at 20 kJ/mol cutoff benchmark. Experimental values were taken from knovel.com.[3] Calculated densities ρ and vaporization enthalpies $\Delta_{vap}H$ are averaged from the last 20 % of the respective simulation (at the melting point established). The lowest temperature at which the box was entirely melted in a span of a simulation is considered the melting point.

Property	Element<br>Model	He	Ne	Ar	Kr	Xe
ρ (g/l)	Exp.	140.6 (3 K)	1239.6	1416.6	2447.7	2973.8
$\Delta_{vap}H$ (kJ/mol)		0.1	1.76	6.54	9.19	12.71
Melting point (K)		none	24.55	83.78	115.78	161.36
ρ (g/l)	WBH	299.8	1300.8	1490.1	2619.5	3179.3
$\Delta_{vap}H$ (kJ/mol)		0.775	2.418	7.795	11.629	16.254
Melting point (K)		9	28	86	135	189
ρ (g/l)	GBH	287.2	1290.5	1459.3	2563.3	3146.0
$\Delta_{vap}H$ (kJ/mol)		0.519	2.000	7.114	10.615	14.804
Melting point (K)		8	27	90	141	198
ρ (g/l)	LJ 12-6	14.1 (3 K)	997.7	1488.2	2614.4	3186.6
$\Delta_{vap}H$ (kJ/mol)		0.024	0.384	6.509	9.872	14.081
Melting point (K)		none	4	77	123	175
ρ (g/l)	LJ 14-7	289.0	1286.3	1455.1	2559.5	3130.7
$\Delta_{vap}H$ (kJ/mol)		0.520	1.890	7.042	10.500	15.019
Melting point (K)		8	25	88	138	195

Table S28: Properties at the melting point of pure noble gases in the liquid phase, simulations of force fields trained with the SAPT/TZ 20 at kJ/mol cutoff benchmark. Experimental values are taken from knovel.com[3] (He-4). Calculated densities ρ and vaporization enthalpies $\Delta_{vap}H$ are averaged from the last 20 % of the respective simulation (at the melting point established). The first temperature at which the box was entirely melted in a span of a simulation is considered the melting point. LJ-14-7 force field were unstable for He (results not available)

Property	Element Model	He	Ne	Ar	Kr	Xe
ρ (g/l)	Exp.	140.6 (3 K)	1239.6	1416.6	2447.7	2973.8
$\Delta_{vap}H$ (kJ/mol)		0.1	1.76	6.54	9.19	12.71
Melting point (K)		none	24.55	83.78	115.78	161.36
ρ (g/l)	WBH	286.6	1199.9	1389.8	2411.3	2942.1
$\Delta_{vap}H$ (kJ/mol)		0.699	1.667	6.328	9.217	13.492
Melting point (K)		8	19	65	99	145
ρ (g/l)	GBH	285.9	1188.0	1366.5	2365.5	2888.6
$\Delta_{vap}H$ (kJ/mol)		0.586	1.401	5.712	8.2714	12.410
Melting point (K)		7	19	70	103	148
ρ (g/l)	LJ-12-6	14.2 (3 K)	1043.7	1375.1	2387.4	2944.3
$\Delta_{vap}H$ (kJ/mol)		0.024	0.599	4.767	6.748	11.488
Melting point (K)		none	7	52	80	137
ρ (g/l)	LJ-14-7	N/A	1194.5	1361.5	2373.5	2903.7
$\Delta_{vap}H$ (kJ/mol)			1.361	5.574	8.3106	12.762
Melting point (K)			17	67	102	151

Table S29: Properties at the melting point of pure noble gases in the liquid phase, simulations of force fields trained with the SAPT/QZ 20 at kJ/mol cutoff benchmark. Experimental values were taken from knovel.com[3] Calculated densities ρ and vaporization enthalpies $\Delta_{vap}H$ are averaged from the last 20 % of the respective simulation (at the melting point established). The first temperature at which the box was entirely melted in a span of a simulation is considered the melting point. LJ-14-7 force field were unstable for He (results not available)

	Element	He	Ne	Ar	Kr	Xe
Property	Model					
ρ (g/l)	Exp.	140.6 (3 K)	1239.6	1416.6	2447.7	2973.8
$\Delta_{vap}H$ (kJ/mol)		0.1	1.76	6.54	9.19	12.71
Melting point (K)		none	24.55	83.78	115.78	161.36
ρ (g/l)	WBH	295.2	1269.1	1456.5	2543.4	3113.6
$\Delta_{vap}H$ (kJ/mol)		0.724	2.042	7.312	10.885	16.036
Melting point (K)		8	23	80	126	184
ρ (g/l)	GBH	287.5	1252.2	1429.8	2504.8	3033.5
$\Delta_{vap}H$ (kJ/mol)		0.639	1.773	6.661	10.1256	14.594
Melting point (K)		8	24	84	131	188
ρ (g/l)	LJ-12-6	14.2 (3 K)	1156.1	1452.3	2550.6	3127.8
$\Delta_{vap}H$ (kJ/mol)		0.024	0.836	5.966	9.0195	14.615
Melting point (K)		none	9	70	111	179
ρ (g/l)	LJ-14-7	N/A	1249.0	1443.4	2502.2	3044.9
$\Delta_{vap}H$ (kJ/mol)			1.544	6.706	9.919	14.991
Melting point (K)			21	81	128	190

.

Table S30: Properties at the melting point of pure noble gases in the liquid phase, simulations of force fields trained with the CCSDT(Q)/CBS at 20 kJ/mol cutoff benchmark. Experimental values are taken from knovel.com.[3] Calculated densities ρ and vaporization enthalpies $\Delta_{vap}H$ are averaged from the last 20 % of the respective simulation (at the melting point established). The first temperature at which the box was entirely melted in a span of a simulation is considered the melting point.

	Element	He	Ne	Ar	Kr	Xe
Property	Model					
ρ (g/l)	Exp.	140.6 (3 K)	1239.6	1416.6	2447.7	2973.8
$\Delta_{vap}H$ (kJ/mol)		0.1	1.76	6.54	9.19	12.71
Melting point (K)		none	24.55	83.78	115.78	161.36
ρ (g/l)	WBH	303.7	1333.4	1506.6	2583.4	3141.1
$\Delta_{vap}H$ (kJ/mol)		0.844	2.484	8.065	11.338	15.893
Melting point (K)		10	29	92	133	183
ρ (g/l)	GBH	301.5	1316.0	1466.5	2532.2	3053.0
$\Delta_{vap}H$ (kJ/mol)		0.611	2.182	7.433	10.382	14.216
Melting point (K)		9	29	96	135	188
ρ (g/l)	LJ-12-6	10.9 (3 K)	1268.2	1503.1	2596.4	3136.6
$\Delta_{vap}H$ (kJ/mol)		0.032	1.323	7.470	10.243	14.383
Melting point (K)		none	16	89	126	178
ρ (g/l)	LJ-14-7	292.4	1306.8	1466.6	2525.1	3059.5
$\Delta_{vap}H$ (kJ/mol)		0.5386	2.016	7.296	10.226	14.382
Melting point (K)		8	28	92	132	186

7 Force Field Parameters

Table S31: Force field parameters from fitting a Lennard-Jones 12-6 potential to quantum chemistry data at the CCSD(T)/CBS level of theory. Energy cut-off 20 kJ/mol for repulsion and 10% of the well-depth at the long range. Rules: ϵ:10 σ:10

Pair		ϵ	σ	RMSE
	kJ/mol	Å	kJ/mol	
He-He	fit	9.61395e-12	16.1265	0.6448
He-Ne	fit	1.87327e-11	16.948	0.3514
	comb	7.87491e-07	7.01048	0.4246
He-Ar	fit	0.00340541	4.1852	0.2680
	comb	3.09094e-06	7.35328	0.7880
He-Kr	fit	8.42191e-05	6.01282	0.3272
	comb	3.73923e-06	7.56551	1.3768
He-Xe	fit	0.00155908	5.0995	0.3030
	comb	4.47427e-06	7.88697	1.9414
Ne-Ne	fit	0.0645043	3.04759	0.2655
Ne-Ar	fit	0.295911	3.18618	0.2130
	comb	0.253183	3.19661	0.4456
Ne-Kr	fit	0.297027	3.34656	0.2596
	comb	0.306285	3.28887	0.8702
Ne-Xe	fit	0.345719	3.54111	0.2343
	comb	0.366493	3.42862	1.3487
Ar-Ar	fit	0.993756	3.35292	0.1423
Ar-Kr	fit	1.18891	3.45721	0.1778
	comb	1.20218	3.44969	0.1988
Ar-Xe	fit	1.38854	3.63211	0.1592
	comb	1.4385	3.59627	0.4469
Kr-Kr	fit	1.45433	3.54926	0.2124
Kr-Xe	fit	1.80418	3.70737	0.1885
	comb	1.74021	3.70007	0.2938
Xe-Xe	fit	2.0823	3.85728	0.2883
RMSE	fit			0.2931
RMSE	comb			0.9781

Table S32: Force field parameters from fitting a Lennard-Jones 8-6 potential to quantum chemistry data at the CCSD(T)/CBS level of theory. Energy cut-off 20 kJ/mol for repulsion and 10% of the well-depth at the long range. Rules: ϵ:10 σ:20

Pair		ϵ	σ	RMSE
	kJ/mol	Å	kJ/mol	
He-He	fit	0.0284724	3.50495	0.1485
He-Ne	fit	0.19032	3.17744	0.0680
	comb	0.120186	3.36345	0.5020
He-Ar	fit	0.321617	3.6229	0.0846
	comb	0.190702	3.73338	0.7432
He-Kr	fit	0.327431	3.83352	0.0896
	comb	0.225418	3.86633	1.1138
He-Xe	fit	0.33199	4.13291	0.0926
	comb	0.263996	4.08442	1.2774
Ne-Ne	fit	0.507321	3.20891	0.1471
Ne-Ar	fit	0.721623	3.63285	0.2042
	comb	0.804978	3.60957	0.2447
Ne-Kr	fit	0.782468	3.79786	0.2119
	comb	0.951519	3.75128	0.3199
Ne-Xe	fit	0.789947	4.05244	0.2184
	comb	1.11436	3.98181	0.6054
Ar-Ar	fit	1.27728	3.93682	0.3078
Ar-Kr	fit	1.50452	4.0603	0.3452
	comb	1.5098	4.05699	0.3463
Ar-Xe	fit	1.63116	4.2706	0.3608
	comb	1.76818	4.25648	0.3812
Kr-Kr	fit	1.78464	4.17044	0.3983
Kr-Xe	fit	2.03074	4.35946	0.4249
	comb	2.09007	4.35991	0.4362
Xe-Xe	fit	2.44777	4.53421	0.5153
RMSE	fit			0.2775
RMSE	comb			0.6827

Table S33: Force field parameters from fitting a Buckingham potential to quantum chemistry data at the CCSD(T)/CBS level of theory. Energy cut-off 20 kJ/mol for repulsion and 10% of the well-depth at the long range. Rules: A:10 C_6:19 b:19

Pair		A	C_6	b	RMSE
	kJ/mol	kJ/mol Å^6	1/Å	kJ/mol	
He-He	fit	41746.5	127.923	4.38631	0.0102
He-Ne	fit	115771	251.216	4.47503	0.0091
	comb	125618	231.518	4.47493	0.6201
He-Ar	fit	182807	849.594	3.91128	0.0155
	comb	226540	387.461	4.05583	0.2568
He-Kr	fit	179706	1262.41	3.67721	0.0183
	comb	245306	420.676	3.89533	0.2204
He-Xe	fit	192648	1909.39	3.43723	0.0197
	comb	273729	449.524	3.70195	0.5649
Ne-Ne	fit	377994	540.123	4.56626	0.0224
Ne-Ar	fit	552765	1771.74	4.02708	0.0213
	comb	681675	1265.61	4.13458	0.2705
Ne-Kr	fit	536972	2596.2	3.80716	0.0283
	comb	738143	1472.21	3.96943	0.4014
Ne-Xe	fit	565917	3883.66	3.58117	0.0331
	comb	823669	1669.78	3.77058	0.5000
Ar-Ar	fit	1.22933e+06	5747.68	3.76134	0.0283
Ar-Kr	fit	1.29022e+06	8431.87	3.60285	0.0394
	comb	1.33117e+06	8053.06	3.61784	0.0853
Ar-Xe	fit	1.48094e+06	12731.4	3.43417	0.0450
	comb	1.48541e+06	10957.5	3.44452	0.3664
Kr-Kr	fit	1.44144e+06	12084.8	3.48241	0.0582
Kr-Xe	fit	1.73784e+06	18625	3.33724	0.0620
	comb	1.60845e+06	17748.2	3.31861	0.1599
Xe-Xe	fit	1.79482e+06	28573.3	3.1661	0.0923
RMSE	fit				0.0401
RMSE	comb				0.3833

Table S34: Force field parameters from fitting a modified Buckingham potential to quantum chemistry data at the CCSD(T)/CBS level of theory. Energy cut-off 20 kJ/mol for repulsion and 10% of the well-depth at the long range. Rules: ϵ:10 σ:20 γ:14

Pair		ϵ	σ	γ	RMSE
	kJ/mol	Å	-	kJ/mol	
He-He	fit	0.0949851	3.00294	13.1718	0.0102
He-Ne	fit	0.175302	3.0486	13.6426	0.0091
	comb	0.179699	3.06167	13.6638	0.3327
He-Ar	fit	0.254922	3.51222	13.7373	0.0155
	comb	0.330266	3.43181	13.6454	0.1269
He-Kr	fit	0.270469	3.71193	13.6496	0.0183
	comb	0.397631	3.57535	13.5401	0.2834
He-Xe	fit	0.260278	4.00718	13.7736	0.0197
	comb	0.471882	3.80421	13.4769	0.3622
Ne-Ne	fit	0.339965	3.11823	14.2386	0.0224
Ne-Ar	fit	0.545881	3.50927	14.1321	0.0213
	comb	0.624817	3.47706	14.217	0.1675
Ne-Kr	fit	0.603488	3.6732	13.9845	0.0283
	comb	0.752262	3.6171	14.0931	0.2742
Ne-Xe	fit	0.617438	3.91447	14.0184	0.0331
	comb	0.892735	3.84116	14.019	0.5448
Ar-Ar	fit	1.14835	3.77405	14.1955	0.0283
Ar-Kr	fit	1.37687	3.89793	14.0436	0.0394
	comb	1.38257	3.89392	14.0724	0.0447
Ar-Xe	fit	1.54362	4.09721	14.0705	0.0450
	comb	1.64075	4.08949	13.9987	0.0824
Kr-Kr	fit	1.66458	4.00683	13.9534	0.0582
Kr-Xe	fit	1.97227	4.18708	13.9733	0.0620
	comb	1.97541	4.19226	13.8822	0.0640
Xe-Xe	fit	2.34429	4.36259	13.8124	0.0923
RMSE	fit				0.0401
RMSE	comb				0.2741

Table S35: Force field parameters from fitting a Wang-Buckingham potential to quantum chemistry data at the CCSD(T)/CBS level of theory. Energy cut-off 20 kJ/mol for repulsion and 10% of the well-depth at the long range. Rules: ϵ:10 σ:20 γ:21

Pair		ϵ	σ	γ	RMSE
	kJ/mol	Å	-	kJ/mol	
He-He	fit	0.105665	3.04261	13.7171	0.0231
He-Ne	fit	0.182348	3.09531	14.3728	0.0228
	comb	0.190111	3.10583	14.3673	0.3333
He-Ar	fit	0.255966	3.56665	14.598	0.0358
	comb	0.339923	3.46714	14.6609	0.1408
He-Kr	fit	0.27566	3.76751	14.4693	0.0410
	comb	0.409573	3.60886	14.5867	0.2845
He-Xe	fit	0.264724	4.0683	14.5976	0.0418
	comb	0.486319	3.8362	14.555	0.3800
Ne-Ne	fit	0.342046	3.16658	15.1196	0.0383
Ne-Ar	fit	0.531886	3.55698	15.2546	0.0571
	comb	0.611586	3.51616	15.463	0.1831
Ne-Kr	fit	0.58914	3.72282	15.0898	0.0674
	comb	0.7369	3.65417	15.376	0.2882
Ne-Xe	fit	0.600317	3.96583	15.171	0.0719
	comb	0.874981	3.87638	15.3389	0.5183
Ar-Ar	fit	1.09353	3.80735	15.8308	0.0820
Ar-Kr	fit	1.31241	3.93045	15.6944	0.1014
	comb	1.31759	3.926	15.7375	0.1038
Ar-Xe	fit	1.46885	4.12636	15.8685	0.1102
	comb	1.56448	4.12065	15.6977	0.1258
Kr-Kr	fit	1.58757	4.03788	15.6459	0.1301
Kr-Xe	fit	1.87821	4.21269	15.8628	0.1399
	comb	1.88505	4.22259	15.6068	0.1432
Xe-Xe	fit	2.23827	4.39241	15.568	0.1867
RMSE	fit				0.0897
RMSE	comb				0.2808

Table S36: Force field parameters from fitting a generalized Buckingham potential to quantum chemistry data at the CCSD(T)/CBS level of theory. Energy cut-off 20 kJ/mol for repulsion and 10% of the well-depth at the long range. Rules: ϵ:10 σ:20 γ:14 δ:10

Pair		ϵ	σ	γ	δ	RMSE
	kJ/mol	Å	-	-	kJ/mol	
He-He	fit	0.0751445	2.82862	12.4563	40	0.0100
He-Ne	fit	0.181196	3.02514	13.8476	11.5104	0.0072
	comb	0.161715	2.9755	13.3414	20.6272	0.0663
He-Ar	fit	0.2607	3.45293	13.7182	14.6247	0.0114
	comb	0.298275	3.35732	13.2316	21.4707	0.0834
He-Kr	fit	0.273098	3.64428	13.618	15.1664	0.0123
	comb	0.360787	3.50091	13.0276	22.2465	0.1373
He-Xe	fit	0.261852	3.92562	13.6765	16.1694	0.0124
	comb	0.428935	3.73423	12.8963	22.5361	0.2464
Ne-Ne	fit	0.348017	3.10915	14.5488	10.637	0.0172
Ne-Ar	fit	0.561476	3.47872	14.227	12.4779	0.0051
	comb	0.641901	3.46368	14.3939	11.072	0.1328
Ne-Kr	fit	0.621944	3.63332	14.012	13.0856	0.0069
	comb	0.77643	3.59908	14.1105	11.472	0.1804
Ne-Xe	fit	0.638841	3.86538	13.9354	13.8295	0.0084
	comb	0.923087	3.82093	13.9307	11.6214	0.4056
Ar-Ar	fit	1.18395	3.75752	14.2455	11.5248	0.0094
Ar-Kr	fit	1.42584	3.87764	13.9773	11.8811	0.0102
	comb	1.43209	3.87351	13.9733	11.9412	0.0325
Ar-Xe	fit	1.6001	4.07849	13.8987	11.9609	0.0135
	comb	1.70259	4.06724	13.8003	12.0966	0.0669
Kr-Kr	fit	1.73222	3.98295	13.7207	12.3726	0.0179
Kr-Xe	fit	2.04946	4.16838	13.6468	12.1962	0.0194
	comb	2.05942	4.16687	13.5597	12.5337	0.0281
Xe-Xe	fit	2.44841	4.33585	13.4059	12.6969	0.0324
RMSE	fit					0.0145
RMSE	comb					0.1766

Table S37: Force field parameters from fitting a Buffered 14-7 potential to quantum chemistry data at the CCSD(T)/CBS level of theory. Energy cut-off 20 kJ/mol for repulsion and 10% of the well-depth at the long range. Rules: ϵ:10 σ:20 γ:11 δ:11

Pair		ϵ	σ	γ	δ	RMSE
	kJ/mol	Å	-	-	kJ/mol	
He-He	fit	0.0744636	2.99042	0.130176	0.0271173	0.0083
He-Ne	fit	0.152872	3.04073	0.140799	2.30727e-19	0.0246
	comb	0.15597	3.04011	0.0837142	0.151072	0.2015
He-Ar	fit	0.238478	3.50405	0.148524	9.33766e-24	0.0122
	comb	0.295014	3.43486	0.135879	0.0363957	0.1504
He-Kr	fit	0.247358	3.70401	0.149189	4.17511e-19	0.0127
	comb	0.356061	3.60044	0.163389	0.0135586	0.2927
He-Xe	fit	0.238308	3.99837	0.143362	9.66891e-25	0.0099
	comb	0.422604	3.83979	0.17036	0.0135586	0.3169
Ne-Ne	fit	0.326692	3.08822	0.0372521	0.275027	0.0315
Ne-Ar	fit	0.540817	3.49161	0.101364	0.0846136	0.0141
	comb	0.617932	3.47273	0.0894173	0.16035	0.0578
Ne-Kr	fit	0.598316	3.65969	0.120064	0.0577353	0.0158
	comb	0.745799	3.63496	0.116927	0.137513	0.1044
Ne-Xe	fit	0.618595	3.92555	0.160371	8.89341e-21	0.0102
	comb	0.885178	3.87019	0.123898	0.137513	0.3714
Ar-Ar	fit	1.16881	3.78695	0.141583	0.045674	0.0025
Ar-Kr	fit	1.40614	3.92409	0.166471	0.0252766	0.0028
	comb	1.41066	3.92476	0.169092	0.022837	0.0099
Ar-Xe	fit	1.57785	4.146	0.192227	0.00122985	0.0021
	comb	1.6743	4.12906	0.176064	0.022837	0.1080
Kr-Kr	fit	1.70257	4.05353	0.196601	1.70937e-21	0.0059
Kr-Xe	fit	2.01524	4.24564	0.20485	2.71419e-25	0.0083
	comb	2.02076	4.24592	0.203573	1.122e-17	0.0331
Xe-Xe	fit	2.39841	4.4223	0.210545	2.24384e-17	0.0207
RMSE	fit					0.0146
RMSE	comb					0.2036

Table S38: Force field parameters from fitting a Tang-Toennies potential to quantum chemistry data at the CCSD(T)/CBS level of theory. Energy cut-off 20 kJ/mol for repulsion and 10% of the well-depth at the long range. Rules: A:19 b:14 C_6:19 C_8:10 C_{10}:11

Pair		A	b	C_6	C_8	C_{10}	Re	De	RMSE
	-	-	-	-	-	Å	kJ/mol	kJ/mol	
He-He	fit	307610	12.1839	0.56171	2.49144e-08	2.94982	2.97397	0.0913674	0.0042
He-Ne	fit	543646	13.309	1.28296	0.716667	4.00382e-08	3.04541	0.173047	0.0080
	comb	499035	12.9465	0.878599	8.22014e-05	1.47491	3.04541	0.173047	0.3739
He-Ar	fit	483192	12.8139	1.00553	8.1397e-11	1.8579	3.49363	0.249118	0.0063
	comb	397828	12.5665	0.789996	7.33947e-13	2.16386	3.49363	0.249118	0.1725
He-Kr	fit	449958	12.6516	0.932619	1.51589e-16	2.17958	3.68043	0.260345	0.0063
	comb	321532	12.2585	0.69185	2.7015e-11	2.69023	3.68043	0.260345	0.5231
He-Xe	fit	489972	12.68	0.814223	9.52245e-13	2.45517	3.96724	0.248871	0.0064
	comb	278967	12.0501	0.620343	7.09135e-10	3.08213	3.96724	0.248871	0.8133
Ne-Ne	fit	946002	13.9455	1.56471	0.271212	2.00022e-11	3.09039	0.351817	0.0220
Ne-Ar	fit	632996	13.1715	0.920076	0.673379	0.96822	3.4968	0.533239	0.0034
	comb	696750	13.4409	1.35943	2.42155e-09	0.688949	3.4968	0.533239	0.1459
Ne-Kr	fit	526446	12.8418	0.981783	8.61128e-17	2.06378	3.66508	0.572622	0.0044
	comb	528070	13.0419	1.14429	8.91321e-08	1.21532	3.66508	0.572622	0.1630
Ne-Xe	fit	506288	12.7484	0.813581	1.65595e-09	2.47806	3.92596	0.566583	0.0055
	comb	440980	12.7764	0.99555	2.33969e-06	1.60722	3.92596	0.566583	0.2209
Ar-Ar	fit	534444	13.0023	1.19205	2.16212e-17	1.3779	3.75716	1.19198	0.0080
Ar-Kr	fit	433161	12.702	1.09137	1.64089e-17	1.77849	3.89316	1.38679	0.0098
	comb	418399	12.651	1.01401	7.95829e-16	1.90427	3.89316	1.38679	0.0336
Ar-Xe	fit	397980	12.5779	1.01849	1.22938e-13	2.00795	4.1043	1.53329	0.0094
	comb	356108	12.4154	0.889207	2.08902e-14	2.29617	4.1043	1.53329	0.0386
Kr-Kr	fit	336422	12.3349	0.873073	2.92928e-14	2.43065	4.01116	1.67507	0.0121
Kr-Xe	fit	327377	12.3049	0.916426	1.66149e-19	2.43141	4.20484	1.92292	0.0139
	comb	290972	12.1214	0.772681	7.68925e-13	2.82254	4.20484	1.92292	0.0542
Xe-Xe	fit	254147	11.922	0.68866	2.0184e-11	3.21443	4.36571	2.35245	0.0201
RMSE	fit								0.0108
RMSE	comb								0.3474

Table S39: Force field parameters from fitting a Lennard-Jones 12-6 potential to quantum chemistry data at the CCSDT(Q)/CBS level of theory. Energy cut-off 20 kJ/mol for repulsion and 10% of the well-depth at the long range. Rules: ϵ:10 σ:10

Pair		ϵ	σ	RMSE
	kJ/mol	Å	kJ/mol	
He-He	fit	1.08264e-11	16.0045	0.6368
He-Ne	fit	0.135218	2.69107	0.0386
	comb	1.44739e-06	6.71787	0.2220
He-Ar	fit	0.155328	3.14232	0.0989
	comb	3.42625e-06	7.31953	0.5206
He-Kr	fit	0.0525783	3.57413	0.2114
	comb	4.04228e-06	7.55632	0.9516
He-Xe	fit	4.82587e-05	6.79563	0.3199
	comb	4.79268e-06	7.88396	2.0945
Ne-Ne	fit	0.193502	2.81981	0.1345
Ne-Ar	fit	0.508158	3.11181	0.0304
	comb	0.458057	3.07236	0.3374
Ne-Kr	fit	0.27226	3.364	0.2554
	comb	0.540414	3.17175	0.8358
Ne-Xe	fit	0.317877	3.5578	0.2292
	comb	0.640735	3.30928	1.4784
Ar-Ar	fit	1.08431	3.34752	0.0902
Ar-Kr	fit	1.12408	3.46836	0.1957
	comb	1.27927	3.45582	0.3488
Ar-Xe	fit	1.32345	3.64173	0.1753
	comb	1.51674	3.60566	0.2767
Kr-Kr	fit	1.50927	3.56762	0.1420
Kr-Xe	fit	1.81905	3.72594	0.1192
	comb	1.78945	3.72231	0.1500
Xe-Xe	fit	2.12164	3.8837	0.2014
RMSE	fit			0.2384
RMSE	comb			0.9398

Table S40: Force field parameters from fitting a Lennard-Jones 8-6 potential to quantum chemistry data at the CCSDT(Q)/CBS level of theory. Energy cut-off 20 kJ/mol for repulsion and 10% of the well-depth at the long range. Rules: ϵ:10 σ:20

Pair		ϵ	σ	RMSE
	kJ/mol	Å	kJ/mol	
He-He	fit	0.0357061	3.42115	0.1510
He-Ne	fit	0.210957	3.1508	0.0479
	comb	0.133114	3.31908	0.1919
He-Ar	fit	0.316742	3.63366	0.0759
	comb	0.209076	3.69354	0.4126
He-Kr	fit	0.340605	3.8254	0.0909
	comb	0.247038	3.84236	0.7504
He-Xe	fit	0.332512	4.13345	0.0931
	comb	0.288302	4.0719	1.3256
Ne-Ne	fit	0.496252	3.21031	0.1549
Ne-Ar	fit	0.603292	3.65742	0.1342
	comb	0.779444	3.60652	0.2071
Ne-Kr	fit	0.789198	3.79758	0.2188
	comb	0.920967	3.76217	0.3290
Ne-Xe	fit	0.793706	4.05284	0.2201
	comb	1.0748	4.00074	0.7604
Ar-Ar	fit	1.22424	3.93077	0.2636
Ar-Kr	fit	1.50232	4.07015	0.3474
	comb	1.44653	4.06303	0.4191
Ar-Xe	fit	1.62989	4.28128	0.3710
	comb	1.68815	4.27009	0.3740
Kr-Kr	fit	1.70917	4.1872	0.3415
Kr-Xe	fit	1.91462	4.38047	0.3960
	comb	1.99466	4.38297	0.4173
Xe-Xe	fit	2.32784	4.56266	0.4368
RMSE	fit			0.2554
RMSE	comb			0.6121

Table S41: Force field parameters from fitting a Buckingham potential to quantum chemistry data at the CCSDT(Q)/CBS level of theory. Energy cut-off 20 kJ/mol for repulsion and 10% of the well-depth at the long range. Rules: A:10 C_6:19 b:10

Pair		A	C_6	b	RMSE
	kJ/mol	kJ/mol Å^6	1/Å	kJ/mol	
He-He	fit	41765.2	134.089	4.37946	0.0109
He-Ne	fit	148248	239.641	4.58746	0.0032
	comb	135104	237.754	4.50491	0.1510
He-Ar	fit	211105	817.008	3.96726	0.0073
	comb	233175	403.735	4.06824	0.2030
He-Kr	fit	196946	1217.16	3.71411	0.0139
	comb	252371	439.022	3.90859	0.2334
He-Xe	fit	192456	1928.76	3.43464	0.0203
	comb	282869	469.628	3.72777	0.8701
Ne-Ne	fit	437037	532.143	4.63395	0.0091
Ne-Ar	fit	809730	1659.39	4.17146	0.0093
	comb	754282	1251.8	4.18477	0.1634
Ne-Kr	fit	562057	2522.14	3.82777	0.0247
	comb	816377	1454.35	4.02055	0.2429
Ne-Xe	fit	583073	3781.3	3.59528	0.0270
	comb	915034	1647.01	3.83455	0.3021
Ar-Ar	fit	1.30182e+06	5758.18	3.77914	0.0276
Ar-Kr	fit	1.18509e+06	8442.69	3.57238	0.0439
	comb	1.40898e+06	8063.47	3.63083	0.1360
Ar-Xe	fit	1.39571e+06	12655.1	3.41477	0.0488
	comb	1.57926e+06	10953.4	3.46287	0.3295
Kr-Kr	fit	1.52498e+06	12091.1	3.48834	0.0420
Kr-Xe	fit	2.05483e+06	18163.6	3.38155	0.0523
	comb	1.70927e+06	17716.8	3.32697	0.1669
Xe-Xe	fit	1.91583e+06	28422.7	3.17306	0.0671
RMSE	fit				0.0330
RMSE	comb				0.3475

Table S42: Force field parameters from fitting a modified Buckingham potential to quantum chemistry data at the CCSDT(Q)/CBS level of theory. Energy cut-off 20 kJ/mol for repulsion and 10% of the well-depth at the long range. Rules: ϵ:21 σ:13 γ:15

Pair		ϵ	σ	γ	RMSE
	kJ/mol	Å	-	kJ/mol	
He-He	fit	0.101774	2.98942	13.092	0.0109
He-Ne	fit	0.173487	3.03833	13.9382	0.0032
	comb	0.138125	3.13843	13.7259	0.1344
He-Ar	fit	0.247707	3.51255	13.9352	0.0073
	comb	0.14338	3.71639	13.6954	0.1922
He-Kr	fit	0.262576	3.71255	13.7888	0.0139
	comb	0.143649	3.93414	13.6478	0.2634
He-Xe	fit	0.263061	4.00632	13.7602	0.0203
	comb	0.143785	4.21044	13.6793	0.6998
Ne-Ne	fit	0.34739	3.10298	14.3791	0.0091
Ne-Ar	fit	0.53321	3.49816	14.5924	0.0093
	comb	0.470642	3.51979	14.3883	0.0879
Ne-Kr	fit	0.58677	3.67543	14.0687	0.0247
	comb	0.480441	3.69868	14.3544	0.2506
Ne-Xe	fit	0.600582	3.91737	14.0841	0.0270
	comb	0.48559	3.93631	14.4063	0.3112
Ar-Ar	fit	1.16028	3.77042	14.2489	0.0276
Ar-Kr	fit	1.34528	3.91141	13.973	0.0439
	comb	1.33568	3.90544	14.1578	0.0683
Ar-Xe	fit	1.50455	4.10923	14.0321	0.0488
	comb	1.45999	4.10554	14.1415	0.0877
Kr-Kr	fit	1.62597	4.02616	14.0446	0.0420
Kr-Xe	fit	1.91366	4.19894	14.1989	0.0523
	comb	1.86708	4.21343	14.002	0.0676
Xe-Xe	fit	2.26179	4.3895	13.9282	0.0671
RMSE	fit				0.0330
RMSE	comb				0.2826

Table S43: Force field parameters from fitting a Wang-Buckingham potential to quantum chemistry data at the CCSDT(Q)/CBS level of theory. Energy cut-off 20 kJ/mol for repulsion and 10% of the well-depth at the long range. Rules: ϵ:21 σ:13 γ:15

Pair		ϵ	σ	γ	RMSE
	kJ/mol	Å	-	kJ/mol	
He-He	fit	0.111493	3.0299	13.6695	0.0243
He-Ne	fit	0.170731	3.0676	15.2511	0.0108
	comb	0.149865	3.16674	14.5508	0.1226
He-Ar	fit	0.248413	3.55426	15.0081	0.0199
	comb	0.15689	3.72161	14.8705	0.1779
He-Kr	fit	0.262027	3.76567	14.7269	0.0331
	comb	0.157277	3.93804	14.7871	0.1760
He-Xe	fit	0.268056	4.06763	14.5727	0.0431
	comb	0.15747	4.21324	14.7979	0.5362
Ne-Ne	fit	0.340927	3.14598	15.4607	0.0320
Ne-Ar	fit	0.517606	3.51858	16.5045	0.0292
	comb	0.461003	3.55175	15.8487	0.1055
Ne-Kr	fit	0.5728	3.72655	15.1535	0.0632
	comb	0.471103	3.73164	15.7869	0.1436
Ne-Xe	fit	0.583742	3.97028	15.2158	0.0650
	comb	0.476376	3.97079	15.8294	0.2527
Ar-Ar	fit	1.11304	3.79394	16.1638	0.0728
Ar-Kr	fit	1.28252	3.94521	15.5704	0.1041
	comb	1.2829	3.92961	16.0547	0.1859
Ar-Xe	fit	1.43211	4.14012	15.7661	0.1128
	comb	1.40249	4.13079	16.045	0.1173
Kr-Kr	fit	1.56557	4.05047	15.9204	0.1019
Kr-Xe	fit	1.81951	4.2158	16.4229	0.1218
	comb	1.79954	4.23835	15.8811	0.1336
Xe-Xe	fit	2.18426	4.41457	15.8075	0.1490
RMSE	fit				0.0776
RMSE	comb				0.2296

Table S44: Force field parameters from fitting a generalized Buckingham potential to quantum chemistry data at the CCSDT(Q)/CBS level of theory. Energy cut-off 20 kJ/mol for repulsion and 10% of the well-depth at the long range. Rules: ϵ:16 σ:18 γ:14 δ:17

Pair		ϵ	σ	γ	δ	RMSE
	kJ/mol	Å	-	-	kJ/mol	
He-He	fit	0.0957698	2.8757	12.7311	23.1298	0.0103
He-Ne	fit	0.175447	3.02714	14.2659	10.7729	0.0007
	comb	0.18025	2.98765	13.5344	20.6634	0.2100
He-Ar	fit	0.249537	3.48561	14.1299	12.1286	0.0020
	comb	0.252061	3.43272	13.372	20.6587	0.1574
He-Kr	fit	0.268909	3.6638	13.8344	13.5295	0.0071
	comb	0.25953	3.62645	13.2638	20.6619	0.1361
He-Xe	fit	0.264267	3.92372	13.6644	16.247	0.0114
	comb	0.248418	3.92094	13.1602	20.6705	0.1037
Ne-Ne	fit	0.354535	3.08493	14.5893	11.7001	0.0033
Ne-Ar	fit	0.541228	3.49044	14.714	11.1995	0.0024
	comb	0.550526	3.48755	14.3711	11.6177	0.0344
Ne-Kr	fit	0.603611	3.63813	14.135	12.8751	0.0084
	comb	0.580156	3.66763	14.2273	11.6732	0.0571
Ne-Xe	fit	0.619539	3.87532	14.0981	13.1577	0.0089
	comb	0.567799	3.94495	14.0906	11.8186	0.2528
Ar-Ar	fit	1.192	3.75876	14.1654	11.5322	0.0082
Ar-Kr	fit	1.3961	3.88843	13.9001	12.0193	0.0134
	comb	1.38526	3.8937	14.0296	11.5898	0.0385
Ar-Xe	fit	1.56248	4.08786	13.8644	12.0981	0.0161
	comb	1.50743	4.11198	13.9002	11.7403	0.1553
Kr-Kr	fit	1.67229	4.01213	13.8988	11.6461	0.0131
Kr-Xe	fit	1.97878	4.18862	13.8122	11.9259	0.0206
	comb	1.90865	4.20804	13.7741	11.7931	0.1096
Xe-Xe	fit	2.32709	4.37296	13.6538	11.9315	0.0238
RMSE	fit					0.0119
RMSE	comb					0.1429

Table S45: Force field parameters from fitting a Buffered 14-7 potential to quantum chemistry data at the CCSDT(Q)/CBS level of theory. Energy cut-off 20 kJ/mol for repulsion and 10% of the well-depth at the long range. Rules: ϵ:10 σ:20 γ:20 δ:11

Pair		ϵ	σ	γ	δ	RMSE
	kJ/mol	Å	-	-	kJ/mol	
He-He	fit	0.0856154	2.97522	0.148184	0.0192404	0.0075
He-Ne	fit	0.174044	3.03258	0.1189	0.0867215	0.0011
	comb	0.171568	3.03133	0.124047	0.063095	0.0046
He-Ar	fit	0.245123	3.49463	0.105357	0.0950257	0.0026
	comb	0.317551	3.44731	0.169057	0.0100876	0.1626
He-Kr	fit	0.252285	3.67192	0.0746788	0.160806	0.0080
	comb	0.375727	3.61235	0.177325	0.00962019	0.2156
He-Xe	fit	0.24018	3.99671	0.143032	1.87314e-19	0.0105
	comb	0.442794	3.85617	0.18335	0.00962019	0.2331
Ne-Ne	fit	0.343813	3.08544	0.0826086	0.10695	0.0052
Ne-Ar	fit	0.537633	3.53016	0.153981	0.0121264	0.0009
	comb	0.636353	3.48938	0.151636	0.0539422	0.1292
Ne-Kr	fit	0.580854	3.65636	0.105564	0.077406	0.0093
	comb	0.752935	3.65073	0.161736	0.0534748	0.1780
Ne-Xe	fit	0.599435	3.908	0.124054	0.0465733	0.0076
	comb	0.887334	3.88991	0.168905	0.0534748	0.3659
Ar-Ar	fit	1.17781	3.8167	0.185756	0.000934821	0.0030
Ar-Kr	fit	1.37667	3.94895	0.186925	0.00293374	0.0052
	comb	1.39358	3.95297	0.192683	0.000467411	0.0191
Ar-Xe	fit	1.53954	4.15688	0.19279	2.7441e-17	0.0056
	comb	1.64234	4.15964	0.197823	0.000467411	0.1854
Kr-Kr	fit	1.64889	4.08044	0.199145	6.68247e-25	0.0047
Kr-Xe	fit	1.94897	4.26407	0.201688	3.5135e-18	0.0101
	comb	1.94322	4.27527	0.203968	2.27936e-14	0.0916
Xe-Xe	fit	2.29009	4.45379	0.208574	4.55872e-14	0.0115
RMSE	fit					0.0070
RMSE	comb					0.1878

Table S46: Force field parameters from fitting a Tang-Toennies potential to quantum chemistry data at the CCSDT(Q)/CBS level of theory. Energy cut-off 20 kJ/mol for repulsion and 10% of the well-depth at the long range. Rules: A:10 b:10 C_6:14 C_8:18 C_{10}:11

Pair		A	b	C_6	C_8	C_{10}	Re	De	RMSE
	-	-	-	-	-	Å	kJ/mol	kJ/mol	
He-He	fit	298681	12.0945	0.496702	6.35413e-09	3.34995	2.97397	0.0913674	0.0034
He-Ne	fit	532455	13.1217	1.26137	6.50816e-08	1.11369	3.04541	0.173047	0.0003
	comb	504041	12.833	0.599231	1.94109e-06	2.16344	3.04541	0.173047	0.1147
He-Ar	fit	514862	12.9171	1.0842	1.32877e-16	1.6276	3.49363	0.249118	0.0011
	comb	365230	12.3934	0.597626	5.69576e-05	2.61162	3.49363	0.249118	0.0725
He-Kr	fit	466617	12.7042	0.956442	1.10905e-15	2.08412	3.68043	0.260345	0.0028
	comb	323171	12.2355	0.593839	6.35413e-09	2.80076	3.68043	0.260345	0.2889
He-Xe	fit	487968	12.6556	0.775631	3.46938e-11	2.57166	3.96724	0.248871	0.0050
	comb	289936	12.0802	0.584089	6.35413e-09	3.05205	3.96724	0.248871	0.8495
Ne-Ne	fit	850596	13.6165	1.2781	1.94111e-06	0.976925	3.09039	0.351817	0.0001
Ne-Ar	fit	729378	13.3599	1.18453	4.43713e-07	1.29122	3.4968	0.533239	0.0005
	comb	616345	13.1501	1.13526	5.68938e-05	1.42511	3.4968	0.533239	0.0292
Ne-Kr	fit	584025	13.0301	1.05441	4.69611e-10	1.72667	3.66508	0.572622	0.0032
	comb	545369	12.9825	1.06415	1.94111e-06	1.61425	3.66508	0.572622	0.0955
Ne-Xe	fit	573885	12.9912	0.976152	2.82338e-15	1.92577	3.92596	0.566583	0.0041
	comb	489283	12.8178	0.95865	1.94111e-06	1.86554	3.92596	0.566583	0.0849
Ar-Ar	fit	446606	12.6997	1.04058	5.69576e-05	1.87329	3.75716	1.19198	0.0034
Ar-Kr	fit	374994	12.4642	0.96928	1.77808e-13	2.16979	3.89316	1.38679	0.0065
	comb	395177	12.5378	0.989249	5.69576e-05	2.06243	3.89316	1.38679	0.0501
Ar-Xe	fit	361556	12.4189	0.914908	1.59659e-10	2.29727	4.1043	1.53329	0.0073
	comb	354537	12.3788	0.908513	5.69576e-05	2.31372	4.1043	1.53329	0.1617
Kr-Kr	fit	349669	12.3781	0.946758	6.94702e-26	2.25157	4.01116	1.67507	0.0060
Kr-Xe	fit	345565	12.3595	0.924731	8.19055e-05	2.34935	4.20484	1.92292	0.0077
	comb	313709	12.221	0.877817	4.17304e-18	2.50287	4.20484	1.92292	0.1240
Xe-Xe	fit	281447	12.0659	0.825416	4.17304e-18	2.75416	4.36571	2.35245	0.0107
RMSE	fit								0.0051
RMSE	comb								0.2974

References

(1) Hirschfelder, J. O.; Curtis, C. F.; Bird, R. B. *Molecular Theory of Gases and Liquids*; Wiley: New York, 1954.

(2) Eckard Bich, R. H.; Vogel, E. Ab initio potential energy curve for the helium atom pair and thermophysical properties of the dilute helium gas. II. Thermophysical standard values for low-density helium. *Mol. Phys.* **2007**, *105*, 3035–3049.

(3) Knovel Corporation, *Knovel Metadatabase*; 2023.
